# Supplementary material for: Single-molecule FRET reveals multiscale chromatin dynamics modulated by HP1α
Source: Nat Commun. 2018 Jan 16;9:235. doi: 10.1038/s41467-017-02619-5 (PMC5770380; doi:10.1038/s41467-017-02619-5)
Supplement: Supplementary file 1 — Supplementary Information [file 41467_2017_2619_MOESM1_ESM.pdf]

Supplementary Figures

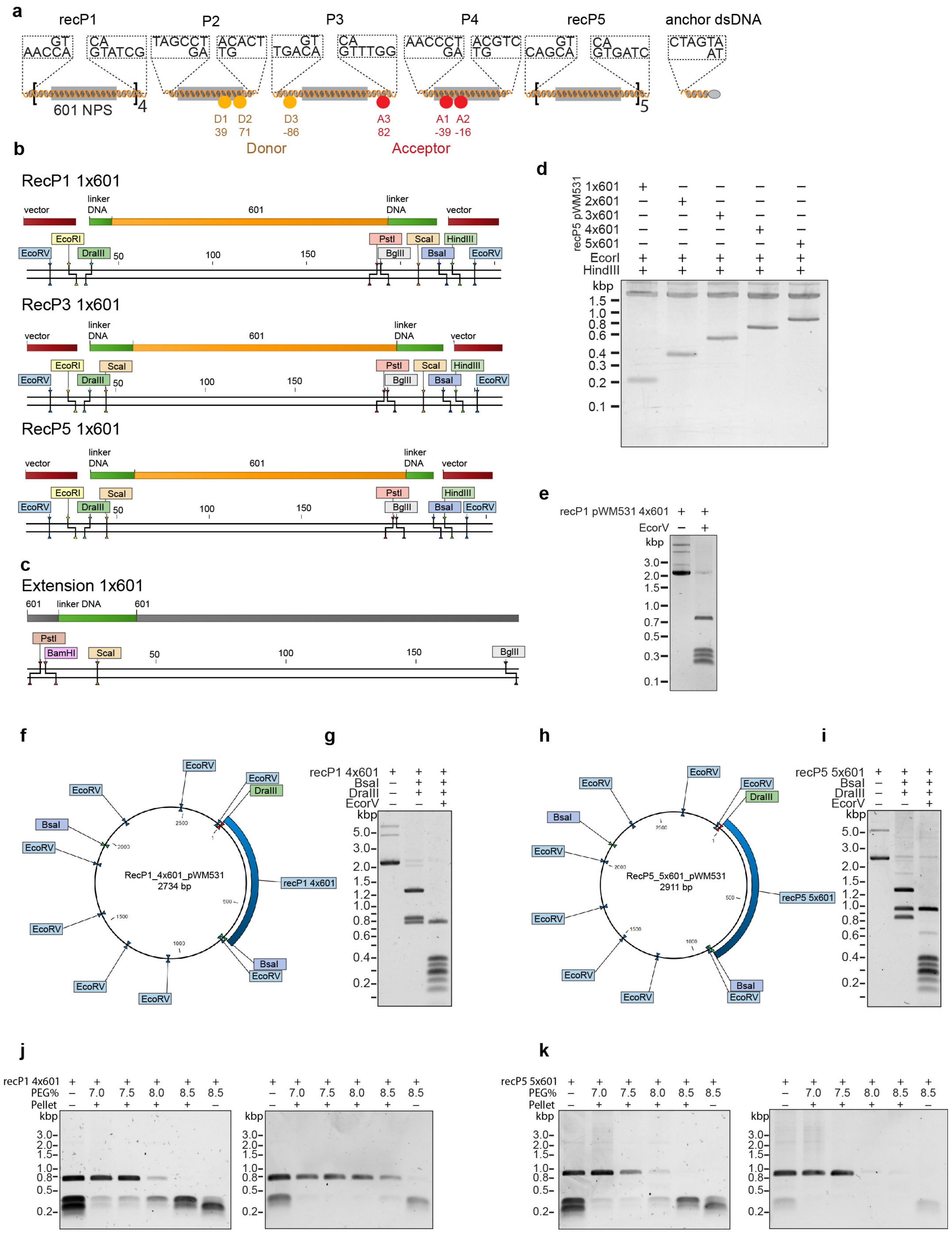

Supplementary Figure 1 | Design, cloning and isolation of recombinant chromatin DNA fragments P1 and P5. (**a**) General design of the library allowing the introduction of FRET pairs into 12-mer chromatin array DNA by a preparative 6-piece ligation of two recombinant (recP1, recP5) and 4 synthetic (P2, P3, P4, anchor) DNA fragments. The anchor contains a biotin for immobilization (grey sphere). Donor (Alexa Fluor 568 (Alexa568) or Alexa Fluor 488, (Alexa488)), Acceptor (Alexa Fluor 647, (Alexa647)). The exact label positions (D1, D2, D3 and A1, A2, and A3; to form DA1, DA2 and DA3) are indicated relative to the 601 sequence, and are compiled in **Supplementary Table 3 & 4**. (**b**) Design of the three recombinant constructs for cloning of chromatin DNA fragments. EcoRV, BsaI and DraIII sites are for DNA fragment excision and plasmid fragmentation. ScaI sites are for quality control of chromatin assemblies to result in individual nucleosomes. PstI and BglII sites are for extension of the array. (**c**) Design of 1x601 nucleosome positioning sequence extension piece. PstI, BamHI and BglII sites are for extension of the array. (**d**) Cloned array DNA pieces of increasing lengths from 1-5x601 excised from the plasmid backbone in recP5. (**e**) Excision of 4x601 DNA from recP1 by EcoRV after modular transfer from other piece. (**f**) Scheme of recP1 4x601 in pWM531 outlining restriction sites for EcoRV, BsaI and DraIII. (**g**) Excision of piece of recP1 4x601 with non-palindromic overhangs by complete digestion first with BsaI and DraIII followed by plasmid backbone fragmentation by EcoRV. (**h**) Scheme of recP5 4x601 in pWM531 outlining restriction sites for EcoRV, BsaI and DraIII. (**i**) Excision of piece of recP1 4x601 with non-palindromic overhangs by complete digestion first with BsaI and DraIII followed by plasmid backbone degradation with EcoRV. (**j-k**) Purification of excised recP1 4x601 (**i**) and recP5 5x601 (**j**) from plasmid backbone fragments by iterative PEG precipitation. For uncropped gels, see **Supplementary Figure 20**.

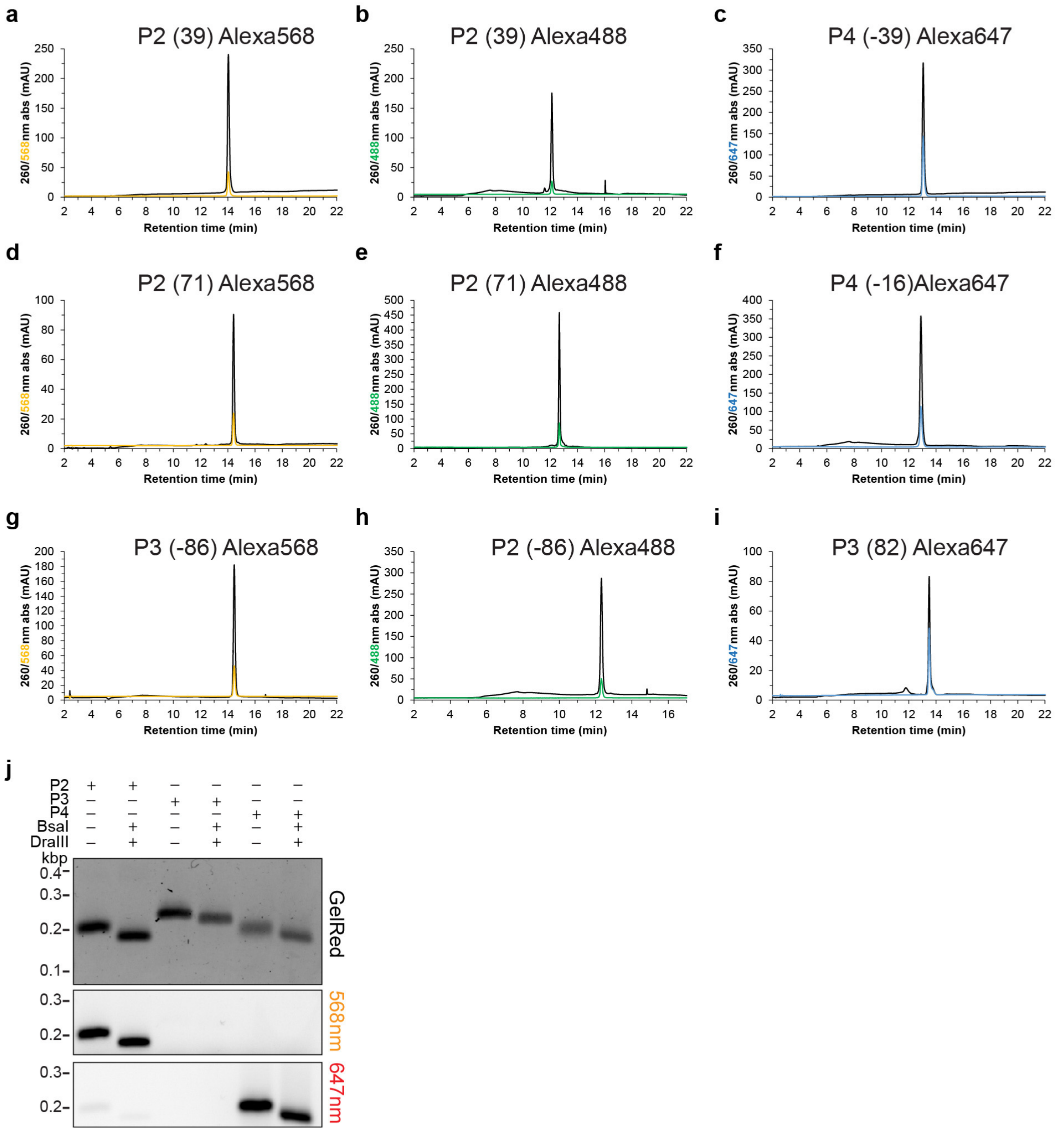

Supplementary Figure 2 | Synthetic oligonucleotide labeling and production of synthetic/PCR-amplified fragments P2-P4. (**a-i**) RP-HPLC analysis of final labeled oligonucleotides for introduction of site-specific labels into PCR pieces. The number in brackets is the final labeling position relative to the nucleosome dyad (see also **Supplementary Tables 1-4**). (**j**) Agarose gel analysis of example PCR-generated pieces P2 (Alexa568 labeled), P3 (unlabeled) and P4 (Alexa647 labeled) before and after digestion with BsaI and DraIII to produce unique non-palindromic cohesive ends. For uncropped gels, see **Supplementary Figure 20**.

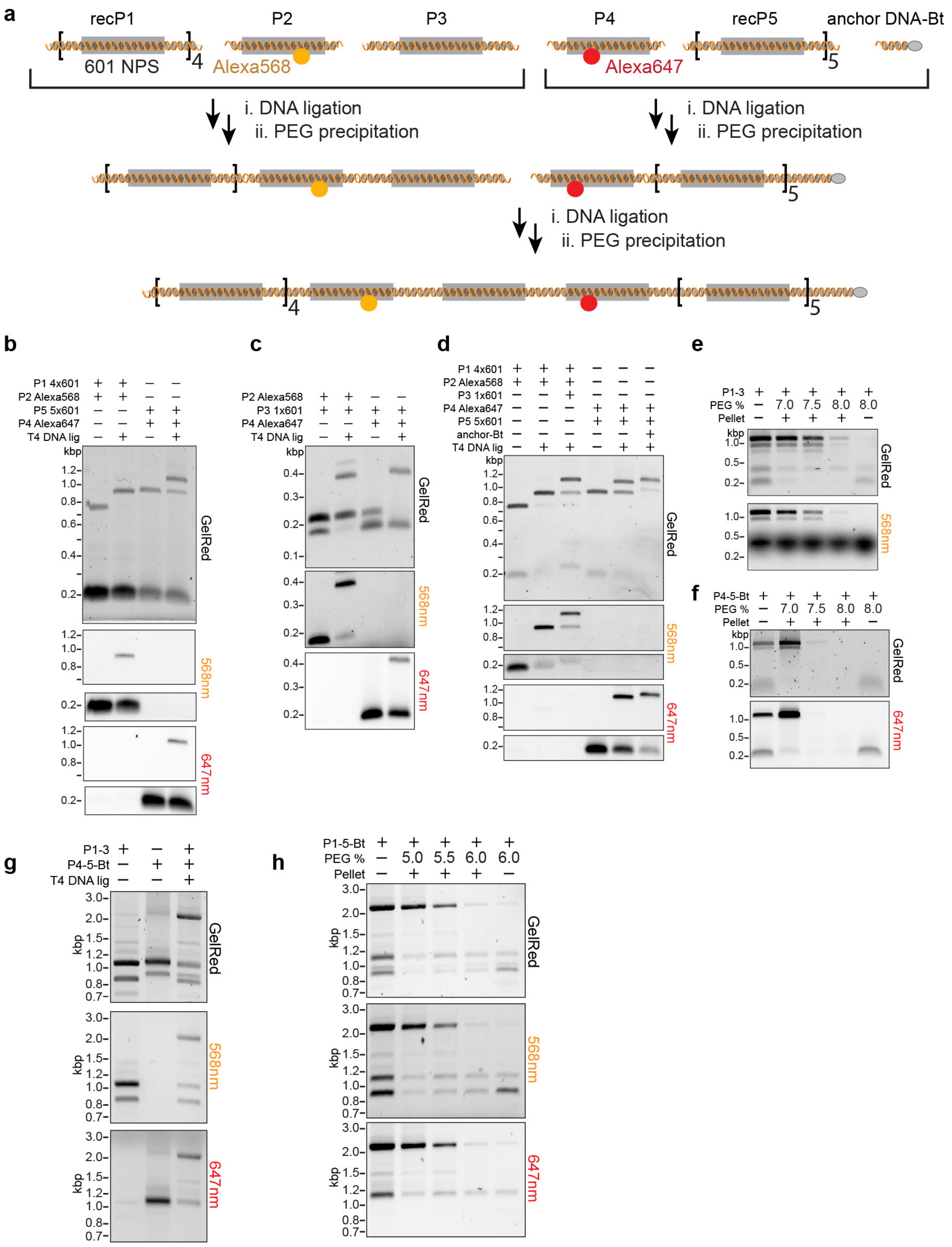

Supplementary Figure 3 | Production of 12x 601 array DNA containing FRET labels. (**a**) Scheme for convergent assembly and purification of 12x array DNA shown for DA1. Pieces recP1 4x601, P2 1x601 and P3 1x601 are ligated and the intermediate 6x601 purified by PEG precipitation from the individual pieces. A similar procedure was used to generate P4 1x601, recP5 5x601 and the dsDNA anchor to produce another 6x601 intermediate and biotinylated (Bt) anchor for TIRF immobilization. The two intermediate 6x601 pieces are

ligated to produce the 12x601 array DNA with internal fluorophores and the Bt-anchor followed by PEG precipitation to separate from the intermediates. (**b-c**) Test ligations to ensure complete digestion of recP1 4x601 and recP5 5x601, P2 1x601, P3 1x601 at key junctions. Complete displacement of the starting pieces upon ligation with excess cognate pieces shows full digestion. (**d**) Samples from large-scale ligations to produce intermediates analyzed to show near-completion of every ligation step. (**e-f**) PEG purification of the 6x601 intermediates to separate from 1x601 pieces that might interfere with final ligation between intermediates. (**g**) Ligation to produce final 12x601 piece displaying the intermediates before and after ligation. (**h**) PEG purification of final 12x601 arrays with <5% remaining of singly labeled and/or DNA lacking the Bt-anchor. For uncropped gels, see **Supplementary Figure 20**.

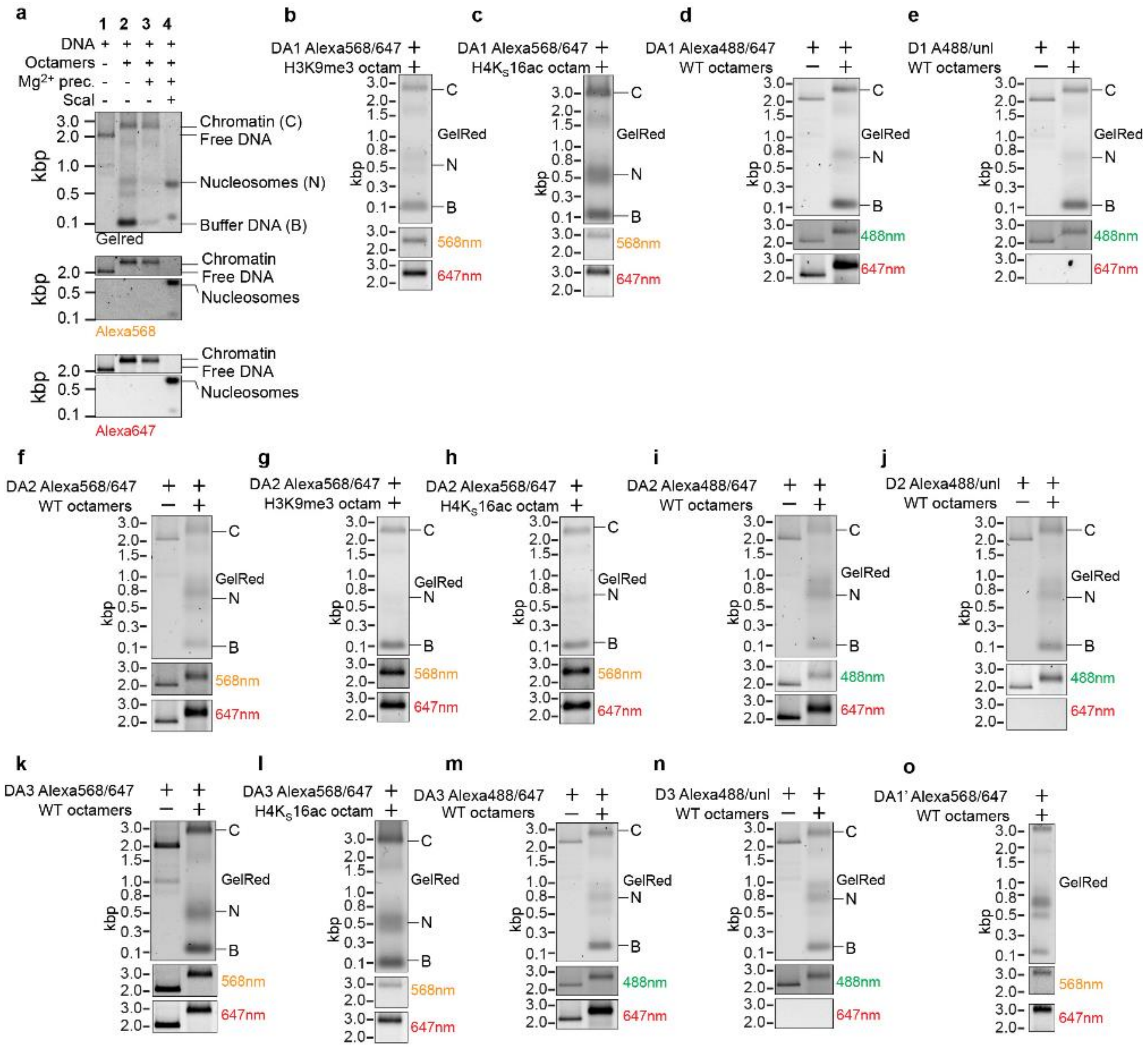

Supplementary Figure 4 | Chromatin formation on DA1-3 fluorescently labeled DNA. (**a**) Analysis of chromatin formation on fluorescently labeled array DNA by agarose gel electrophoresis. Lane 1: Free DA1-labeled array DNA. Lane 2: Assembled chromatin arrays. To avoid overloading array DNA with histone octamers, low-affinity buffer DNA (B) is added, resulting in the formation of a small amount of buffer nucleosomes (N). Lane 3: Chromatin arrays are purified by Mg^{2+} precipitation. Lane 4: Digestion with the restriction enzyme ScaI liberates mononucleosomes. The absence of higher-order aggregates or significant amounts of free DNA demonstrates the saturation of chromatin arrays. (**b**)**,** Formation of DA1 chromatin arrays with Alexa568/647 labels and H3K9me3 containing histone octamers, (**c**)**,** DA1 chromatin arrays with Alexa568/647 labels and $H4K_S16ac$ octamers. (**d**) DA1 chromatin arrays with Alexa Fluor 488 (Alexa488) and Alexa647 labels. **e,** D1 chromatin arrays with an Alexa488 label (Donor-only). (**f**) DA2 chromatin arrays with Alexa568/647 labels

and unmodified histone octamers. (**g**) DA2 chromatin arrays with Alexa568/647 labels and H3K9me3 containing octamers. (**h**) DA2 chromatin arrays with Alexa568/647 labels and H4K_S16ac containing histone octamers. (**i**) DA2 chromatin arrays with Alexa488/647 labels and unmodified histone octamers. (**j**) D2 chromatin arrays with an Alexa488 label (Donor only). (**k**) DA3 chromatin arrays with Alexa568/647 labels and unmodified histone octamers. (**i**) DA3 chromatin arrays with Alexa568/647 labels and H4K_S16ac histone octamers. (**m**) DA3 chromatin arrays with Alexa488/647 labels and unmodified histone octamers. (**n**) D3 chromatin arrays with an Alexa488 label (Donor only). (**o**) DA1’ chromatin arrays with Alexa568/647 with an n, n+1 dye spacing to test FRET in solenoid structures. For uncropped gels, see **Supplementary Figure 20**.

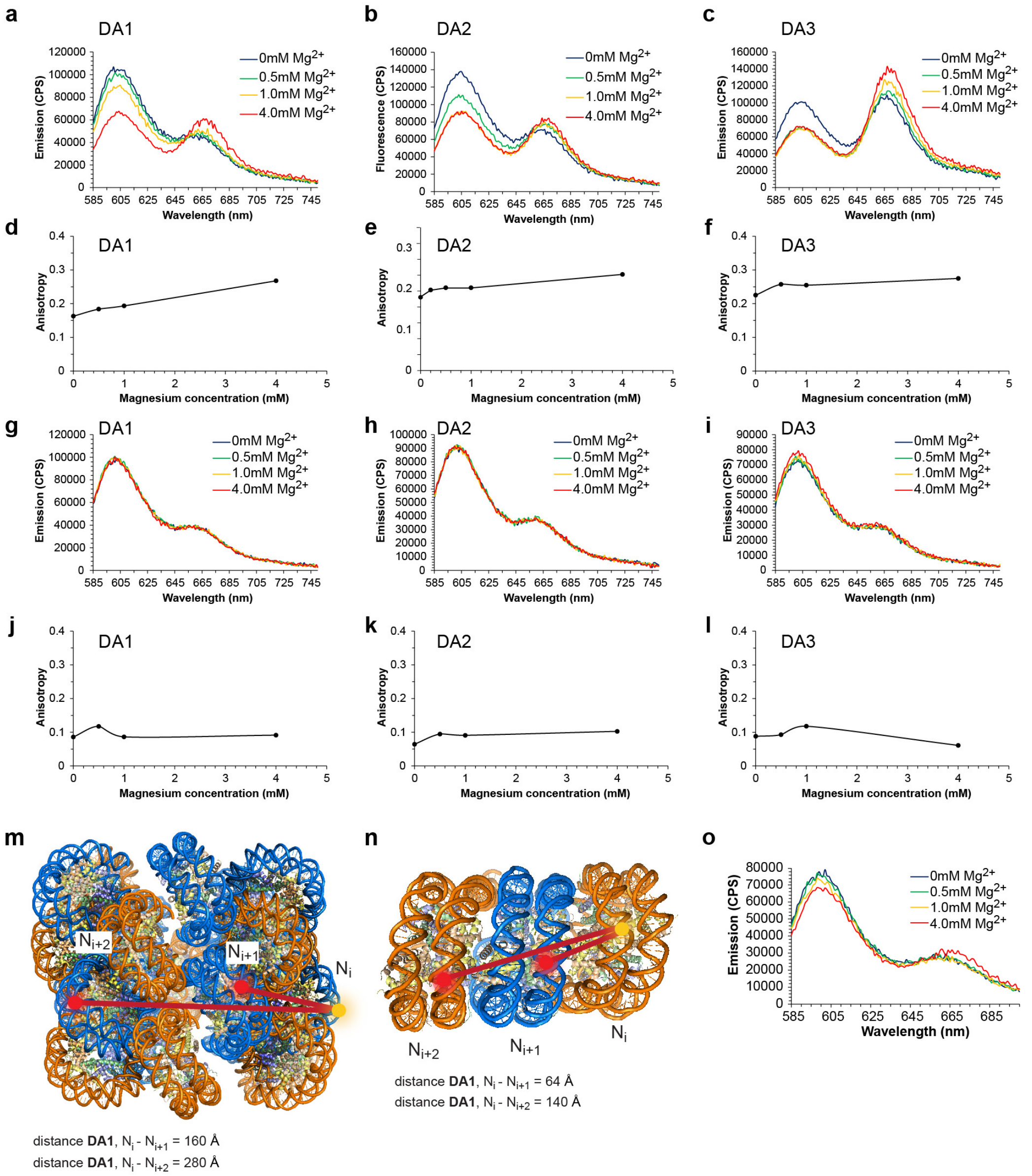

Supplementary Figure 5 | Fluorescence spectra and donor anisotropies in chromatinized and non-chromatinized double-labeled array DNA (Alexa568/647). (**a-c**) Ensemble spectra of chromatin samples upon compaction with magnesium, showing distinct responses dependent on the positions of the FRET pairs. (**d-f**) Anisotropy of the donor in the chromatin arrays at increasing magnesium concentrations. (**g-i**) Spectra of the double-labeled DNA samples in absence of nucleosomes. (**j-l**) Anisotropy of the donor in absence of nucleosomes. (**m**) Alternative chromatin structure (interdigitated solenoid) based on a model from ref. [1], exhibiting inter-dye distances (DA1) outside the FRET detection radius (see also **Supplementary Fig. 8b**). (**n**) Alternative chromatin structure (solenoid) with continuous stacking of nucleosomes, exhibiting inter-dye distances within FRET detection radius only for N_i, N_{i+1} labeling distance. (**o**) Fluorescence data on **DA1'** chromatin array with a N_i,N_{i+1} label configuration, exhibiting minor FRET increase and thus indicating that solenoid structures do not contribute to the measured FRET signal.

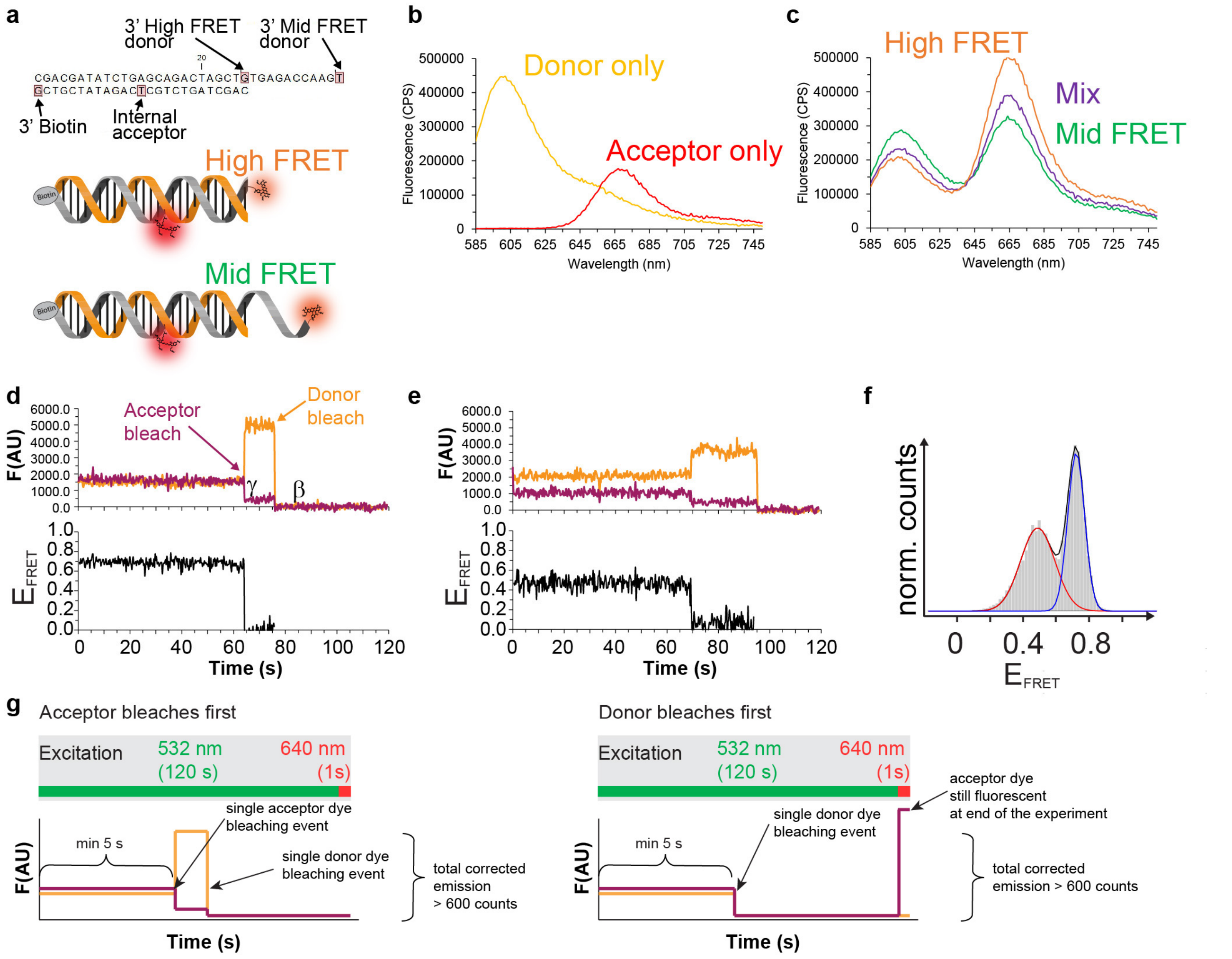

Supplementary Figure 6 | Characterization of FRET pair at the single-molecule level. (See **Supplementary Note**, **step 1**: smTIRF). (**a**) Sequences and locations of fluorophores and biotin on constructs used to calibrate ensemble and smFRET with Alexa Fluor 568 and Alexa Fluor 647. (**b**) Spectra acquired from ensemble FRET by excitation at 575nm from single-stranded oligonucleotides labeled with each of the two respective dyes. (**c**) Spectra from the individual dsDNA constructs showing distance-dependent FRET. (**d**) Schematic of the two annealed dsDNA constructs. (**e**) Trace from high FRET DNA piece at the single-molecule level with TIRF with indications of bleaching events, the relative detection efficiencies γ, between donor and acceptor and the donor bleedthrough to the acceptor β, for calculations of the corresponding FRET efficiencies. (**e**) Trace from mid FRET DNA piece with sm FRET. (**f**) Histograms from mixture between two DNA pieces showing the ability to distinguish between different populations using the FRET pair. (**g**) Trace selection criteria: Shown are the two types of acceptable traces that were used for all smTIRF analyses, as judged by trace length, emission and dye bleaching behavior. For details see **Materials and Methods** or **Supplementary Note**, paragraph **smTIRF measurements.**

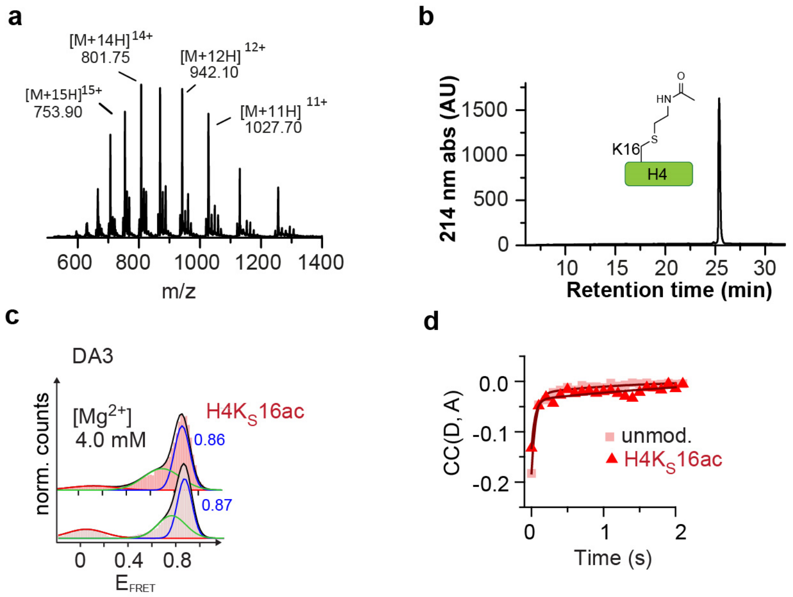

Supplementary Figure 7 | H4K$_S$16ac analytical data and impact in DA3. H4K$_S$16ac is produced by reacting the mutant H4, H4K16C, with N-vinylacetamide in the presence of radical promoter (VA-044) and glutathione[2]. (**a**) MS spectrum of semisynthetic H4K$_S$16ac. (Expected mass: 11211Da, observed mass 11211Da) (**b**) RP-HPLC analysis of H4K$_S$16ac. (**c**) FRET histogram for DA3 at 4mM Mg^{2+} with or without acetylation on H4 K16. (**d**) Donor-acceptor channel cross-correlation analysis of DA1, overlay of data for 4 mM Mg^{2+} for unmodified chromatin, as well as H4K$_S$16ac at 4 mM Mg^{2+}. The fit for H4K$_S$16ac results in a relaxation time t_R = 50 ms. For the percentage of dynamic traces, see **Supplementary Table 6.**

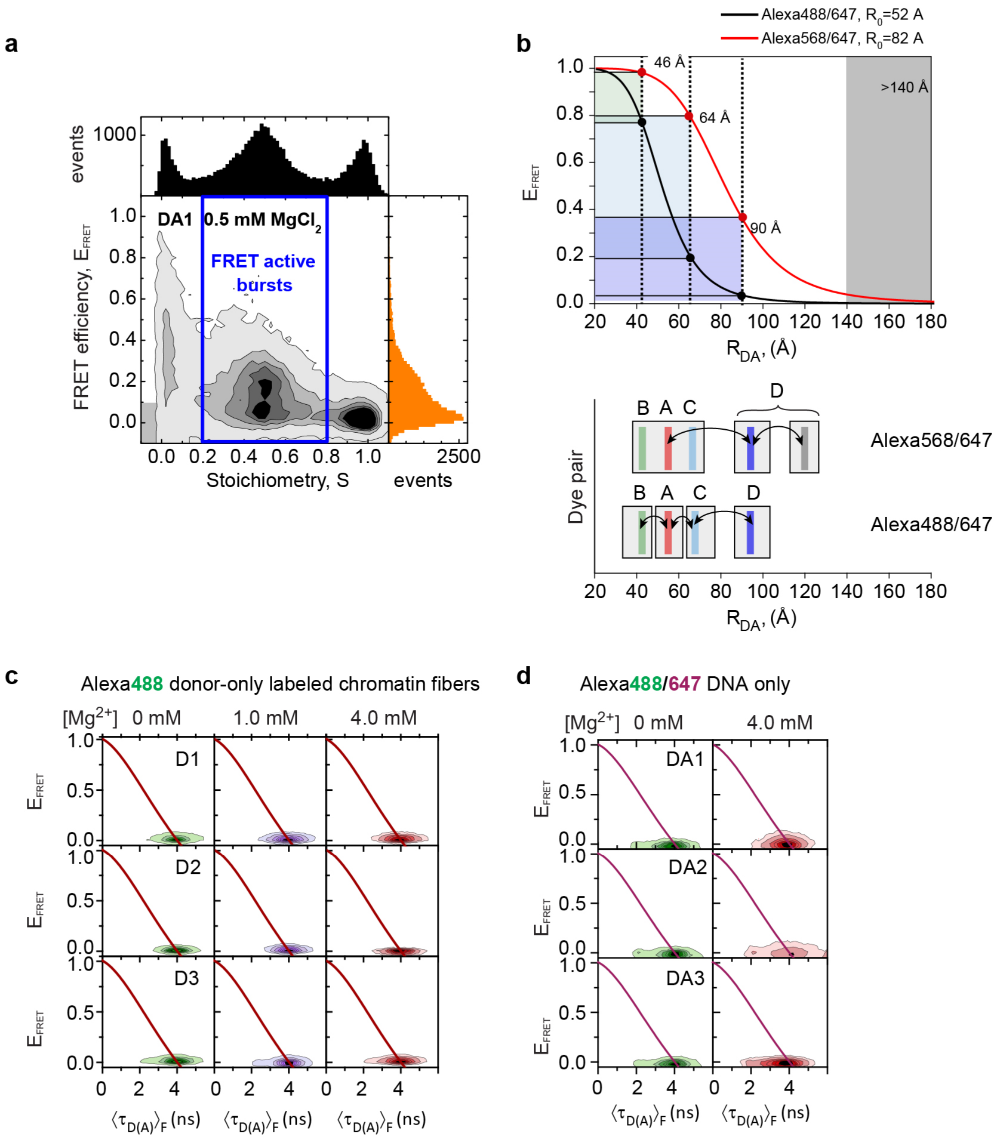

Supplementary Figure 8 | MFD measurements of chromatin arrays DA1-3 with Alexa568/647 and Alexa488/647 labeling schemes. (See **Supplementary Note, step 2**: 2D MFD plots with FRET lines). (**a**) MFD histogram of E_{FRET} vs Stoichiometry for DA1 (Alexa488/647) in 0.5 mM Mg^{2+}. Blue box: Selection of bursts of double-labeled chromatin arrays capable of FRET (FRET active population). (**b**) E_{FRET} as a function of inter-dye distance for the two employed dye-pairs, Alexa568/647 and Alexa488/647. For illustration, representative inter-dye distances and their associated E_{FRET} values observed in DA1 are indicated. Alexa568/647 and Alexa488/647 have different sensitivities: Alexa568/647 allows the detection of long-range dynamics beyond 120 Å, whereas Alexa488/647 enables the investigation of sub-states and their exchange dynamics below 70 Å. (**c**) MFD plots of donor-only, Alexa488-labeled chromatin fibers (D1, D2 and D3). Dark red line: static FRET line. See **Supplementary Methods, step 2, Static and dynamic FRET-lines**. (**d**) MFD plots of Alexa488/647 labeled DNA (DA1, DA2 and DA3), demonstrating the absence of FRET or dynamics without the presence of chromatin.

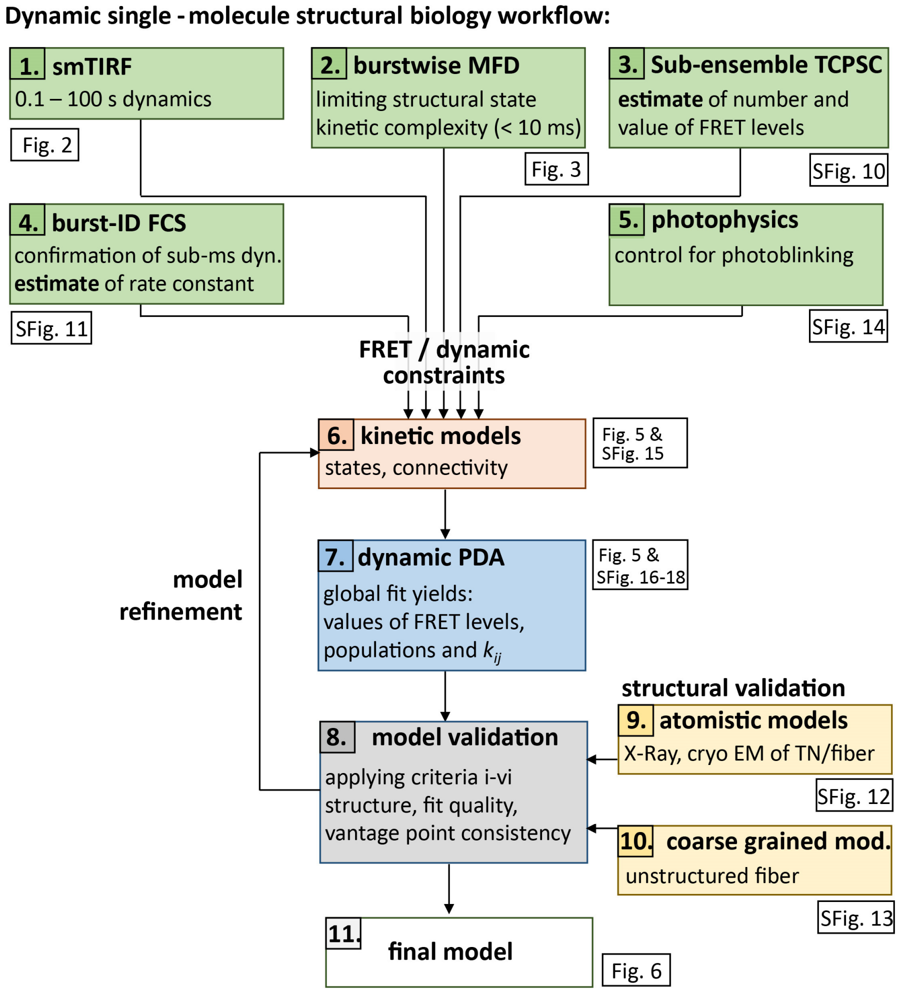

Supplementary Figure 9 | Dynamic single-molecule structural biology workflow. (See **Supplementary Note,** Dynamic structural biology analysis). The workflow is comprised of 11 steps: 5 experimental methods (shaded in green), design of the kinetic model (orange), data analysis with dynamic PDA methods (in blue), validation of the model (gray), structural validation (in yellow) and confirmation of the final model (white). Small boxes: Indicating Figures containing the relevant data, "SFig" refers to Supplementary Figures.

a

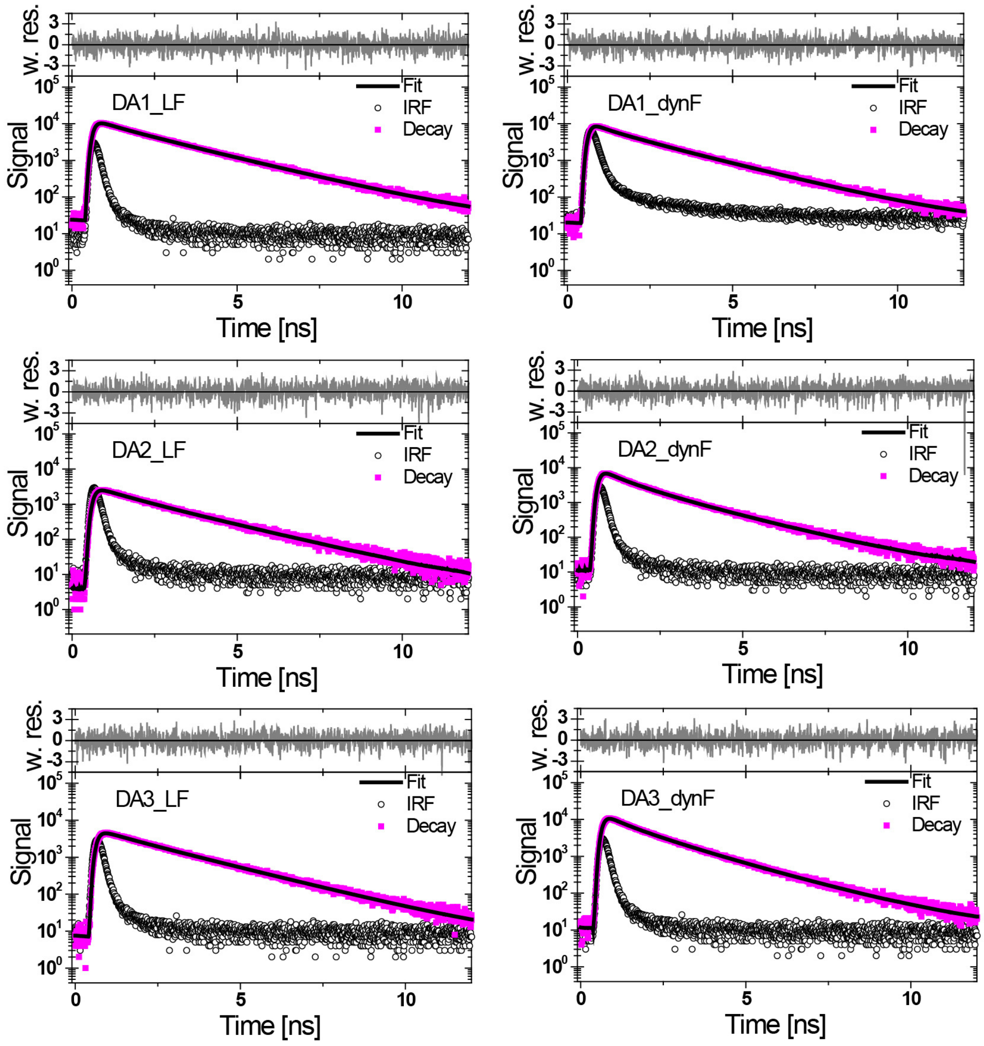

b

subpopulations	$R_{DA,1}$, (Å)	x_1	$R_{DA,2}$, (Å)	x_2	$R_{DA,3}$, (Å)	x_3	χ^2_R
**DA1 LF**	46	0.40			90	0.60	0.98
**DA1 dynFRET**	41	0.42	65	0.18	120	0.40	0.98
**DA2 LF**			52	0.39	128	0.61	1.06
**DA2 dynFRET**	42	0.41	55	0.35	120	0.24	1.15
**DA3 LF**			54	0.24	120	0.76	1.06
**DA3 dynFRET**	40	0.36	57	0.45	120	0.19	1.02

Supplementary Figure 10 | seTCSPC of DA1-3 (Alexa488/647) in 0.5 mM Mg^{2+}. (See **Supplementary Note, step 3**: Sub-ensemble TCSPC). (**a**) Fluorescence decays (magenta) with corresponding fit (black line). Left panel: fits for accumulated LF bursts; right panel: fits for accumulated dynF bursts. In the global fit (see **Supplementray Note, step 3**) the DOnly decay $f_{D(0)}$ was approximated by a single donor fluorescence lifetime ($\tau_{D(0)}$ = 4.1 ns) and the decay of the FRET-population $f_{D(A)}$ with 3 Gaussian distributed distances and the same fixed half-width σ_{DA}= 6 Å. The fit quality is illustrated by weighted residuals (in the upper panel) by χ^2_r. (**b**) Fit results for the LF and dynF populations by eq.(3.3) - (3.4). IRF: Instrument response function.

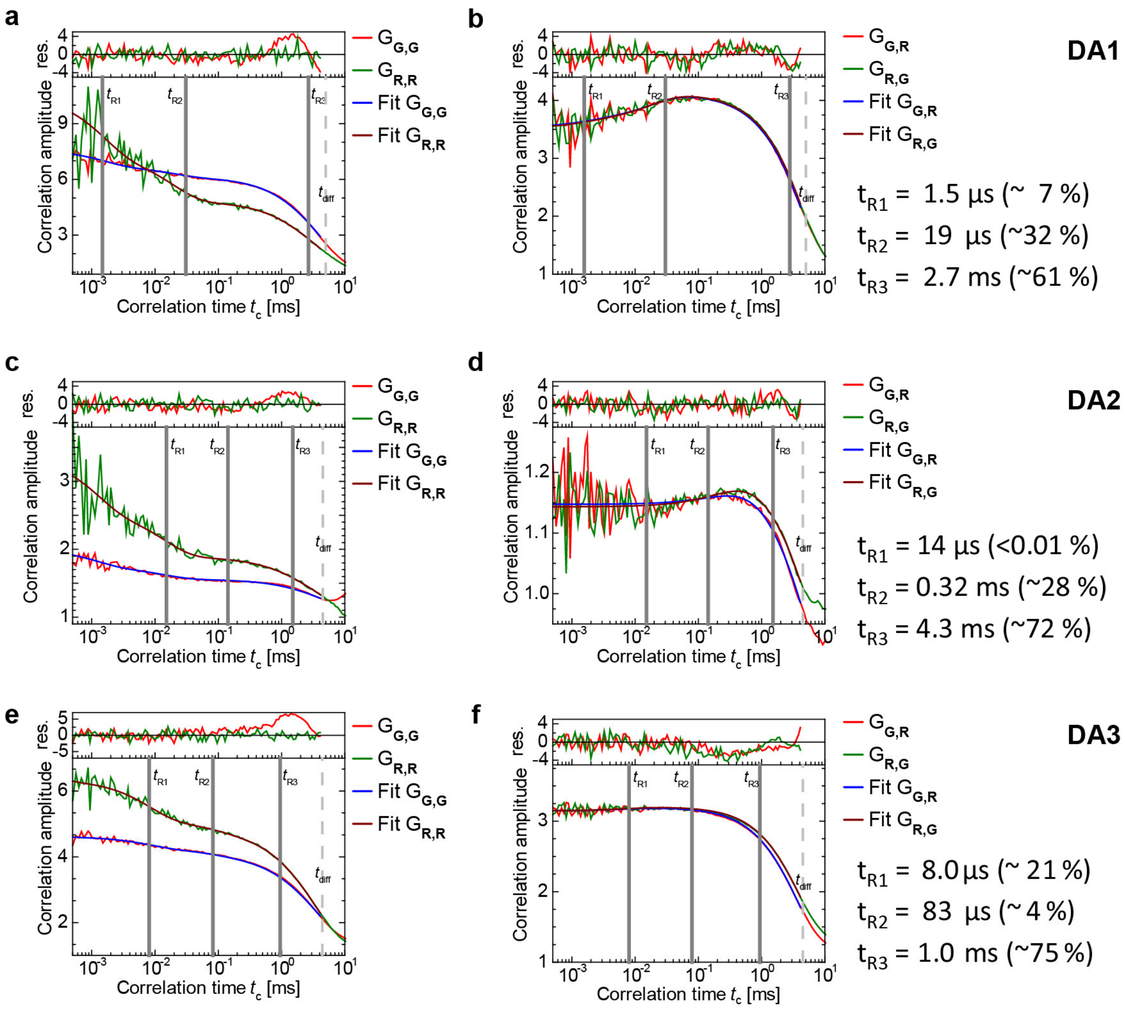

Supplementary Figure 11 | Model-free dynamic analysis by fluorescence correlation functions of arrays DA1-3 (Alex488/647) at 0.75 mM Mg^{2+}. (See **Supplementary Note**, **step 4**: Burst-ID FCS). The analysis by auto- and cross-correlation functions[3] shows FRET related anti-correlated dynamics with three relaxation time t_{R1}, t_{R2}, and t_{R3} in the ms- and sub-ms time range. (**a**) Burst-ID donor-donor ($G_{G,G}$) and acceptor-acceptor ($G_{R,R}$) auto-correlation functions for DA1 chromatin fibers with an additional bunching term in the auto-correlation functions to consider also dye blinking with the corresponding amplitudes $B^{(G)}$ = 0.09 and $B^{(R)}$= 0.27, respectively, and the relaxation time t_B= 44 ns. (**b**) Burst-ID cross-correlation functions $G_{G,R}$ and $G_{R,G}$ of donor-acceptor (G-R) and acceptor-donor (R-G) signal, respectively. The timescales of observed processes are obtained by a global fit of all correlation functions for one FRET pair and are shown on the right. (**c-d**) Auto- and cross-correlation functions for DA2 with the additional bunching amplitudes $B^{(G)}$ = 0.25 and $B^{(R)}$ = 0.33, respectively, and the relaxation time t_B = 1.2 µs. (**e-f**) Auto- and cross-correlation functions for DA3 at with the additional bunching amplitudes $B^{(G)}$ = 0.12 and $B^{(R)}$ = 0.25, respectively, and the relaxation time t_B = 0.13 µs.

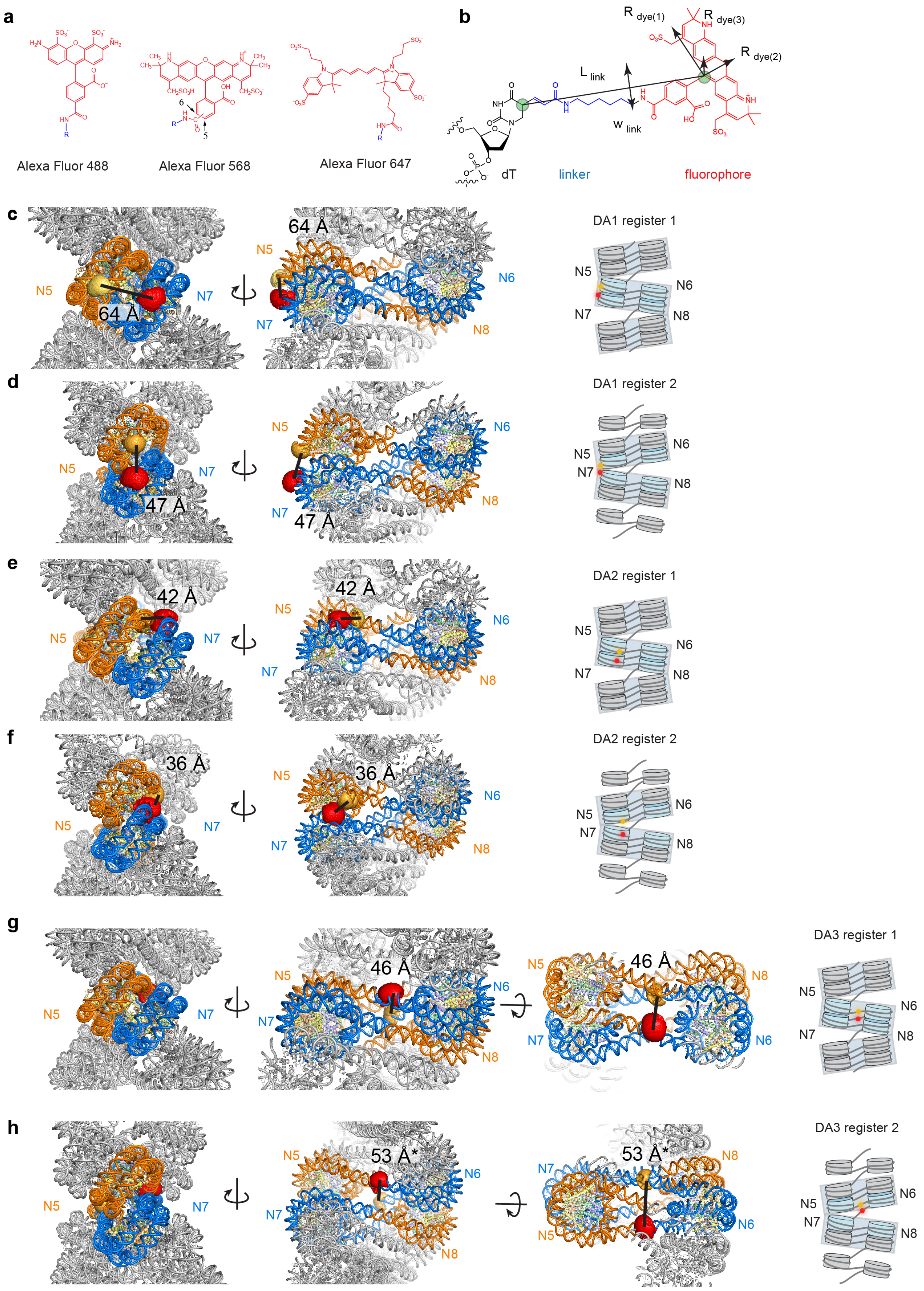
a
Alexa Fluor 488
Alexa Fluor 568
Alexa Fluor 647
b
R dye(1)
R dye(3)
R dye(2)
L link
W link
dT
linker
fluorophore
c
64 Å
DA1 register 1
d
47 Å
DA1 register 2
e
42 Å
DA2 register 1
f
36 Å
DA2 register 2
g
46 Å
DA3 register 1
h
53 Å*
DA3 register 2
N5
N6
N7
N8

Supplementary Figure 12 | Structural models for the compact chromatin state. (See **Supplementary Note**, **step 9**: Structural models of compact chromatin states). For a comparison between measured and calculated distances, see **Supplementary Table 8**. (**a**) Chemical structures of the fluorescent labels Alexa488, Alexa568 and Alexa647. (**b**) Chemical structure of the dye linked to a dT nucleotide. To calculate the dye accessible contact volumes for these dyes, the structure was approximated by an ellipsoid ($R_{dye(1)}$, $R_{dye(2)}$ and $R_{dye(3)}$) connected by a linker of length (L_{link}) and width (w_{link}). Accessible contact volume dye model was used where part of AV which is closer than 3 Å from the macromolecular surface is defined to have higher dye density ρ_{dye}[4]. For the parameters used for the different dyes, see **step 9: FRET positioning and screening calculations**. (**c**) Molecular structure of a compact chromatin array, consisting of a stack of 3 tetranucleosomes (4-4-4, register 1) with DA1-positioned dyes in the central tetranucleosome. The model was produced by fitting nucleosomes into the electron density of the cryoEM structure of a 177-bp nucleosome array, ref. [5]. The inter-dye distance was evaluated using simulated dye accessible contact volumes (ACV)[6]. (**d**) Molecular structure of a chromatin array, consisting of a stack of 2 tetranucleosomes, flanked by two unstacked nucleosomes at each side (2-4-4-2, register 2) with DA1-positioned dyes on the two central tetranucleosomes and inter-dye distance from ACV-calculations. (**e**) Inter-dye distance for DA2 dyes in register 1 compacted arrays. (**f**) Inter-dye distance for DA2 dyes in register 2 compacted arrays. (**g**) Inter-dye distance for DA3 dyes in register 1 compacted arrays. Linker DNA was introduced extending the nucleosomal DNA connecting neighboring nucleosomes. The distance is calculated between the phosphate groups of the modified bases (P-P distance). (**h**) Inter-dye distance for DA3 dyes in register 2 compacted arrays. Linker DNA was introduced extending the nucleosomal DNA connecting neighboring nucleosomes. Shown are calculated P-P distances.

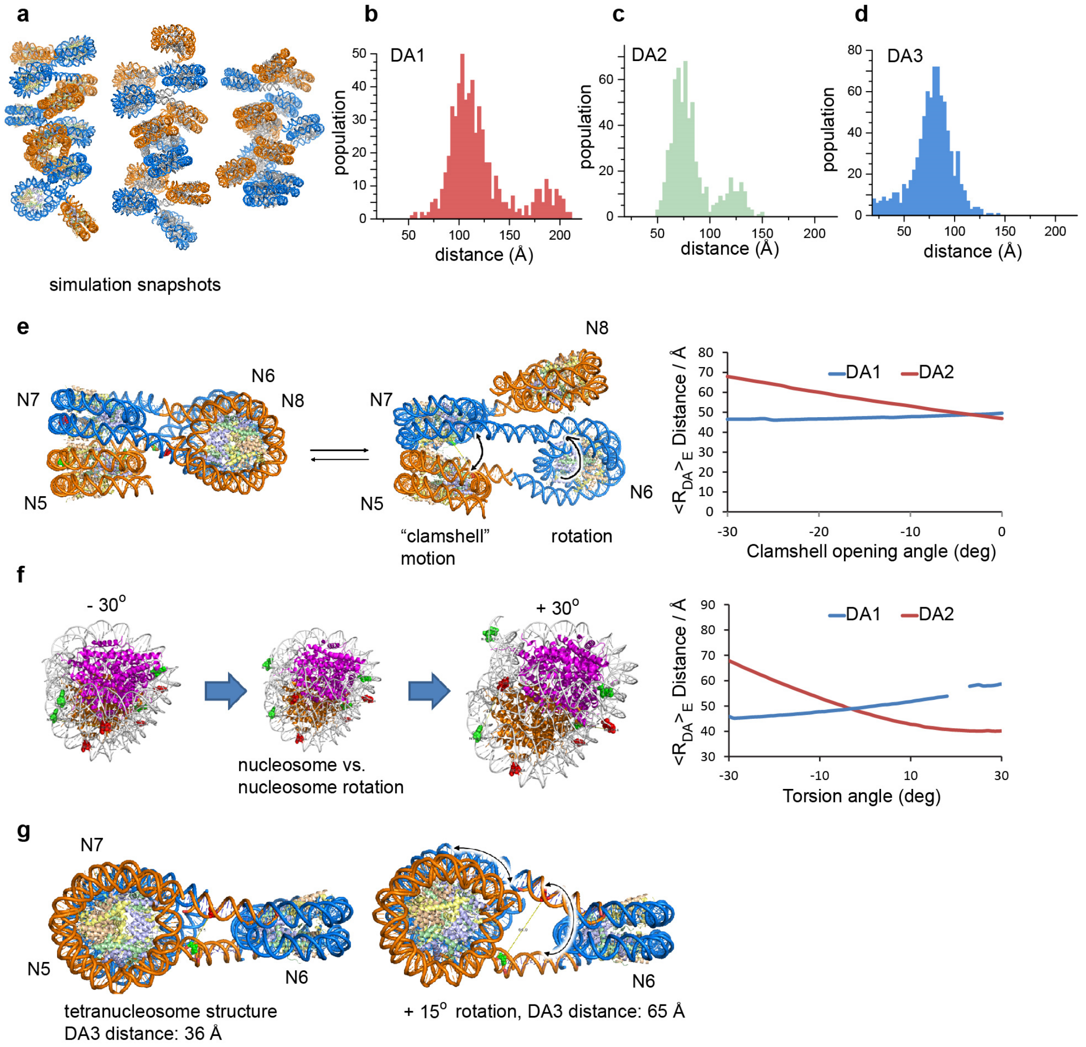

Supplementary Figure 13 | Structural models open and dynamic chromatin states. (See **Supplementary Note**, **step 10**: Structural models of open and dynamic states). (**a**) Representative snapshots from coarse grained simulations of chromatin fibers following ref. [7]. (**b**) Calculated distance distributions between DA1 dye pairs (between phosphate groups of the modified bases, P-P distances) in the open chromatin ensembles for 100 structures with 12 nucleosomes each. (**c**) Calculated P-P distance distributions between DA2 dye pairs in open chromatin ensembles from the same structure set as in b. (**d**) Calculated P-P distance distributions between DA3 dye pairs in open chromatin ensembles for the same structure set as in **b**. (**b**-**d**) Distances are calculated between P atoms of the labeled nucleotide. (**e**) Dependence of DA1 and DA2 FRET averaged inter-dye distance on "clamshell"-type opening of the tetranucleosome interface. DA1 is not sensitive to this mode of motion, in contrast to DA2. (**f**) Dependence of DA1 and DA2 inter-dye distance on rotational motions between two nucleosomes. DA2 shows stronger angular dependency compared to DA1. (**g**) Effect of rotational motion on DA3 FRET averaged inter-dye distance showing that this dye pair is sensitive to the distorted tetranucleosome state (State C in **Fig. 4a**).

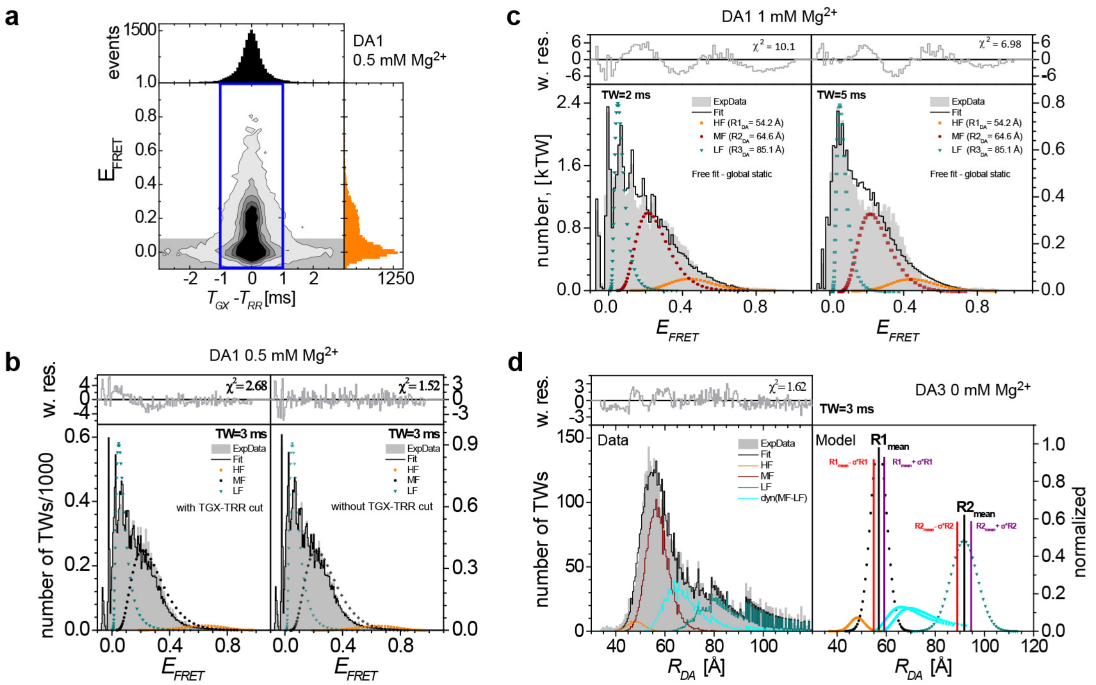

Supplementary Figure 14 | Photobleaching and photoblinking analysis & principles of PDA analysis. (See **Supplementary Note**, **step 5**: Photobleaching and photoblinking analysis, and **step 7**: General description of PDA analysis). (**a**) Example of the photobleaching and photoblinking analysis for for DA1 (Alex488/647) in 0.5 mM Mg^{2+}. FRET efficiency vs T_{GX}-T_{RR} is arranged in a 2D histogram, demonstrating the procedure of the macro-time filter ($|T_{GX}$-$T_{RR}| < 1$ ms). Bursts selected for the macrotime cut are in the blue box. For a definition of the parameter see **Dynamic Analysis, step 5**. (**b**) PDA analysis of the FRET efficiency histograms of the selected bursts (right panel) and un-filtered bursts (left panel). The difference between the two analyses is very small. **Principles of PDA analysis:** (**c**) DA1 (Alexa488/647) in 1 mM Mg^{2+}. A global fit for two TWs (2 ms (left panel) and 5 ms (right panel) using a joint fit model (3 static Gaussian distributed distances) demonstrates that a static model inappropriate. Experimental data histogram is shown in gray, fit in black line and resulting static FRET states in orange, dark cyan and wine. (**d**) Dynamic PDA analysis overview using DA3 (Alexa488/647) at 0 mM Mg^{2+} as an example. Left panel: experimental data histogram is shown in gray and resulting shot-noise limited model distribution as a black line. It is described by the contribution of a High FRET species (HF, orange), medium FRET species (MF, wine), low FRET species (LF, dark cyan) and a dynamic species in a two-state dynamic distribution between MF and LF (cyan line). Right panel: The model distance distribution is given by a sum of static Gaussian-distributed distances (R1, MF: (wine symbols), R2, LF: (dark cyan symbols)) and dynamic mixing between ($R1_{mean}$- $\sigma 1$ and $R2_{mean}$- σ_2) and ($R1_{mean}$+ σ_1 and $R2_{mean}$+ σ_2) distributions (cyan symbols), where $\sigma_i=\sigma*R_i$ and $\sigma=0.06$.

		Selection criteria:					
Connectivity plot	Equations	i. connectivity	ii. state #	iii. parameter boundaries	iv. fit stability	v. goodness of fit	vi. consistency
**DA1**							
1.	$A,C \rightleftarrows D$<br>$A,C \rightleftarrows B$<br>$B \rightleftarrows D$	+	+	−	−	+	−
2.	$A,C \rightleftarrows D$<br>$B \rightleftarrows D$	+	+	+	+	+	+
**DA2**							
1.	$A,C \rightleftarrows D$<br>$A,C \quad B$	−	+	−	−	+	−
2.	$B \rightleftarrows D$<br>$A,C \rightleftarrows D$	+	+	−	−	−	+
3.		−	+	+	+	+	−
4. con-strained to DA1	$A \rightleftarrows C$<br>$C \rightleftarrows D$<br>$A \rightleftarrows D$<br>$B \rightleftarrows D$	+	+	−	−	−	+
5.	$A \rightleftarrows C$<br>$A \rightleftarrows D$<br>$B \rightleftarrows D$	−	+	+	+	+	+
6.	$A \rightleftarrows C$<br>$C \rightleftarrows D$<br>$B \rightleftarrows D$	+	+	+	+	+	+
**DA3**							
1.	$A \rightleftarrows C$<br>$C \rightleftarrows D$<br>$B \rightleftarrows D$	−	−	−	+	+	+
2.	$A,B \rightleftarrows C$<br>$C \rightleftarrows D$<br>$A,B \rightleftarrows D$	+	+	+	−	+	−
3.	$A,B \rightleftarrows D$<br>$C \rightleftarrows D$	−	+	+	+	+	−
4.	$A,B \rightleftarrows C$<br>$C \rightleftarrows D$	+	+	+	+	−	−
5. A - C constrained to DA2	$A \rightleftarrows C$<br>$C \rightleftarrows D$<br>$B \rightleftarrows D$	+	+	+	+	+	+

Supplementary Figure 15 | List of the trial models in PDA analysis for DA1-3. (See **Supplementary Note, step 8**: Validation of kinetic models). The first column represents sketches of applied models for particular FRET dye configuration. The model was evaluated with several selection criteria, see **step 8, Validation of kinetic models**. Cases when criteria meets the model are marked in green, the discrepancy are in red. The model was chosen if all criteria are satisfied.

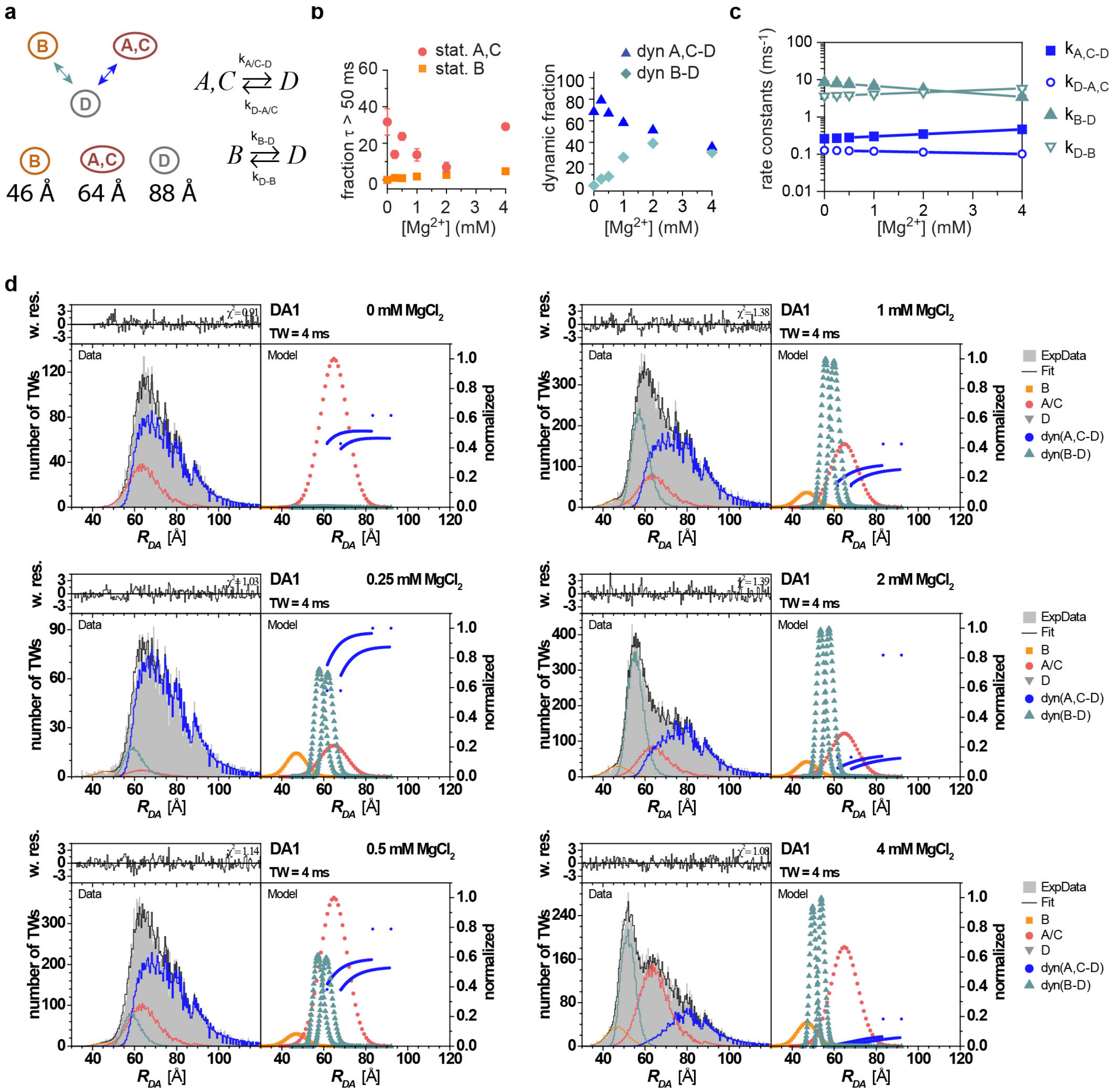

Supplementary Figure 16 | PDA fit of MFD data for DA1 (Alexa488/647). (See **Supplementary Note**, **step 7**: General description of PDA analysis & **step 8**: Validation of kinetic models). (**a**) Minimal dynamic model used to globally fit the experimental data. Distances in Å are given for the states {A,C} (are not differentiated by DA1), B, and D. (**b**) Left panel: Fractions of molecules which appear static on the MFD timescale (10 ms). Right panel: Dynamic fractions – molecule exchanging between the indicated states with rate constants given in **c**. (**c**) Rate constants obtained from the global PDA fit. (**b-c**) Error bars: s.d. between three PDA analyses of datasets comprising a fraction (70%) of all measured data (subsampling). Note that in some cases the error bars are smaller than the symbol size. (**d**) Individual PDA fits of the model given in **c** to the experimental data at the indicated conditions, showing the fit, residuals as well as the underlying static (symbols in red hues and grey) and dynamic (symbols in blue hues) molecular distributions.

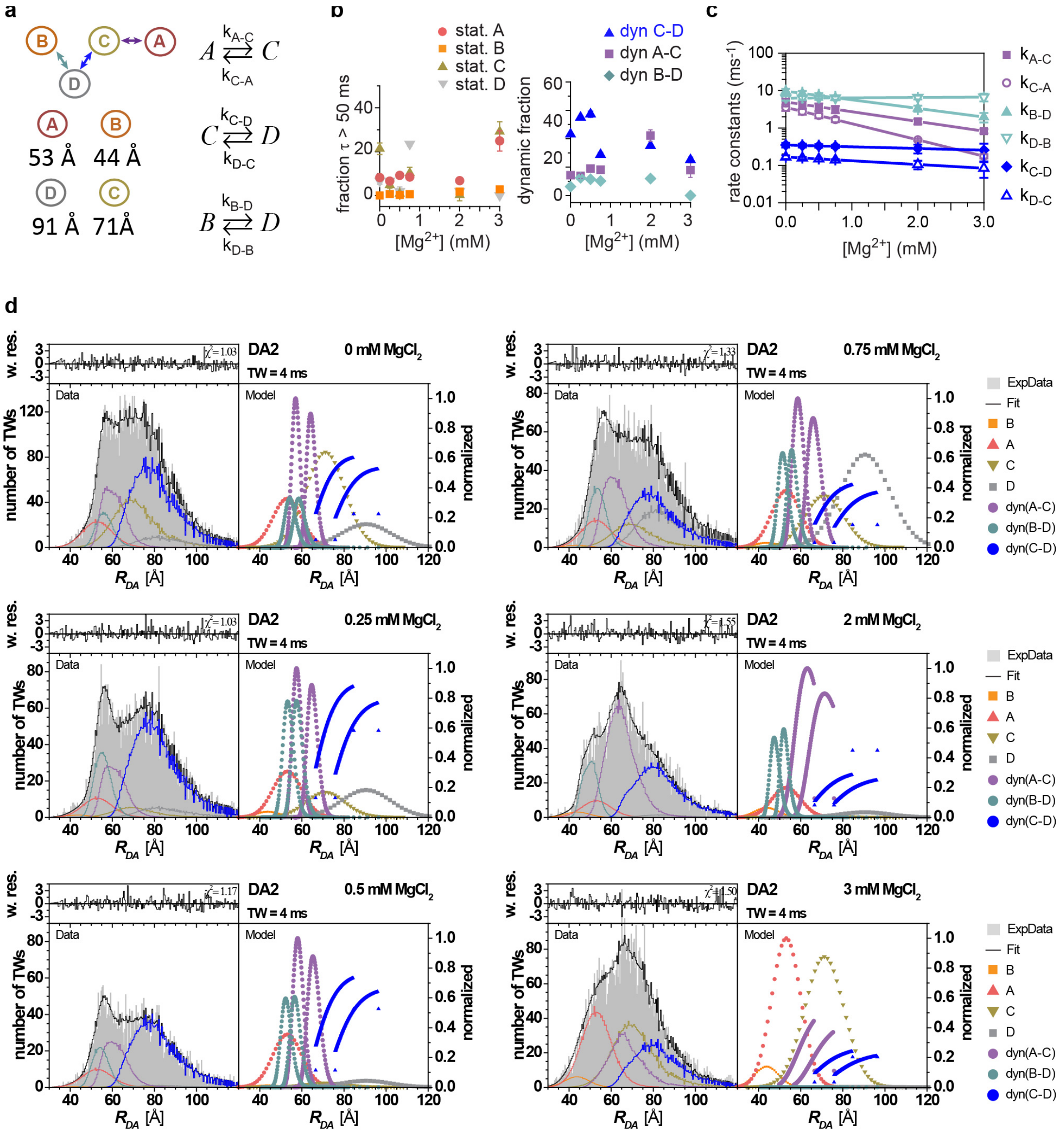

Supplementary Figure 17 | PDA fit of MFD data for DA2 (Alexa488/647). (See **Supplementary Note**, **step 7**: General description of PDA analysis & **step 8**: Validation of kinetic models). (**a**) Minimal dynamic model used to globally fit the experimental data. Distances in Å are given for the states A, B, C and D. (**b**) Left panel: Fractions of molecules which appear static on the MFD timescale (10 ms). Right panel: Dynamic fractions – molecule exchanging between the indicated states with rate constants given in **c**. (**c**) Rate constants obtained from the global PDA fit. (**b-c**) Error bars: s.d. between three PDA analyses of datasets comprising a fraction (70%) of all measured data (subsampling). Note that in some cases the error bars are smaller than the symbol size. (**d**) Individual PDA fits of the model given in **a** to the experimental data at the indicated conditions, showing the fit, residuals as well as the underlying static (symbols in red hues and grey) and dynamic (symbols in blue hues) molecular distributions.

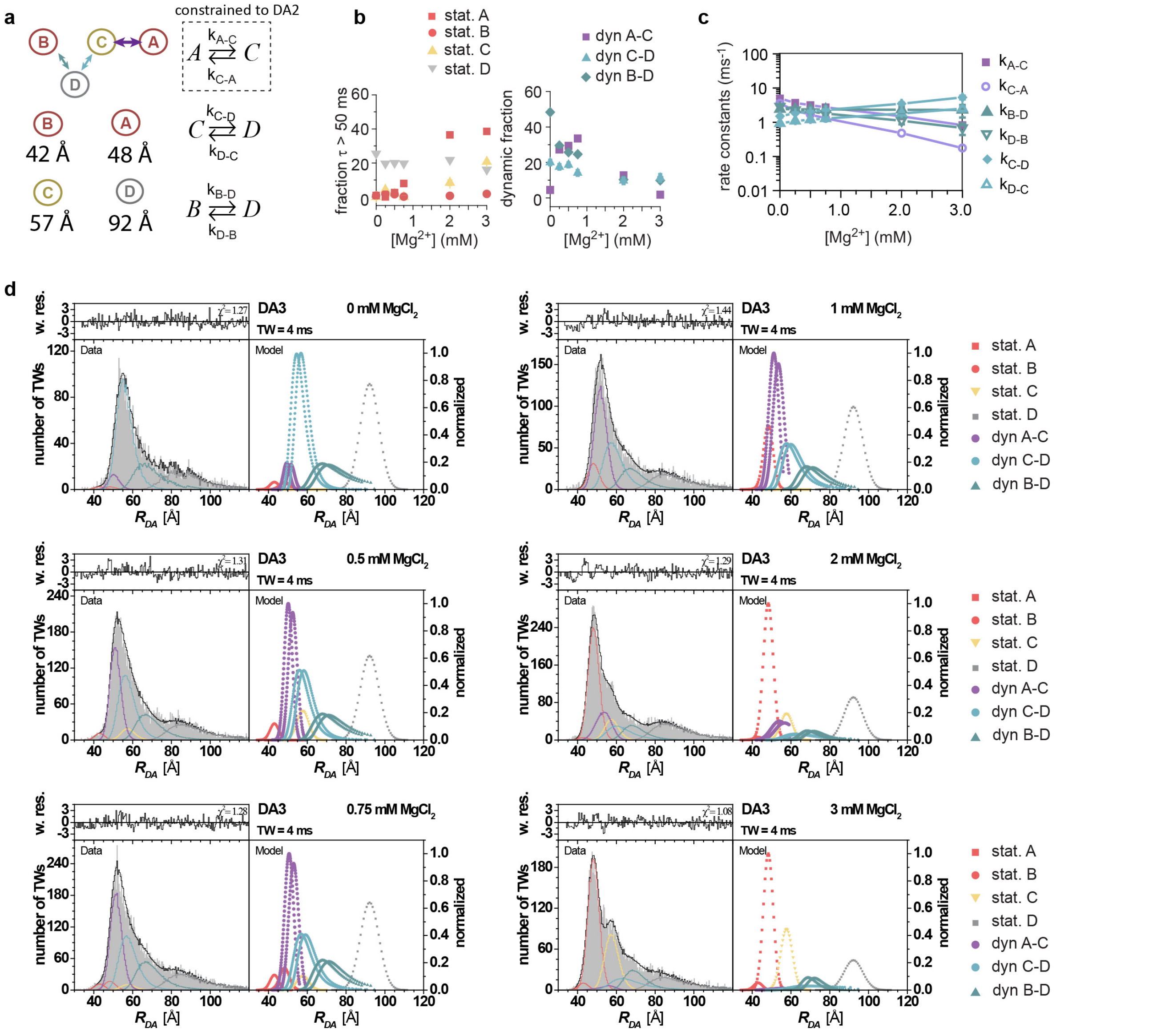

Supplementary Figure 18 | PDA fit of MFD data for DA3 (Alexa488/647). (See **Supplementary Note**, **step 7**: General description of PDA analysis & **step 8**: Validation of kinetic models). (**a**) Minimal dynamic model used to globally fit the experimental data. Distances are given in Å. (**b**) Left panel: Fractions of molecules which appear static on the MFD timescale (10 ms). Right panel: Dynamic fractions – molecule exchanging between the indicated states with rate constants given in **c**. (**c**) Rate constants obtained from the global PDA fit. (**b-c**) Error bars: s.d. between three PDA analyses of datasets comprising a fraction (70%) of all measured data (subsampling). Note that in some cases the error bars are smaller than the symbol size. (**d**) Individual PDA fits of the model given in **a** to the experimental data at the indicated conditions, showing the fit, residuals as well as the underlying static (symbols in red hues and grey) and dynamic (symbols in blue hues) molecular distributions.

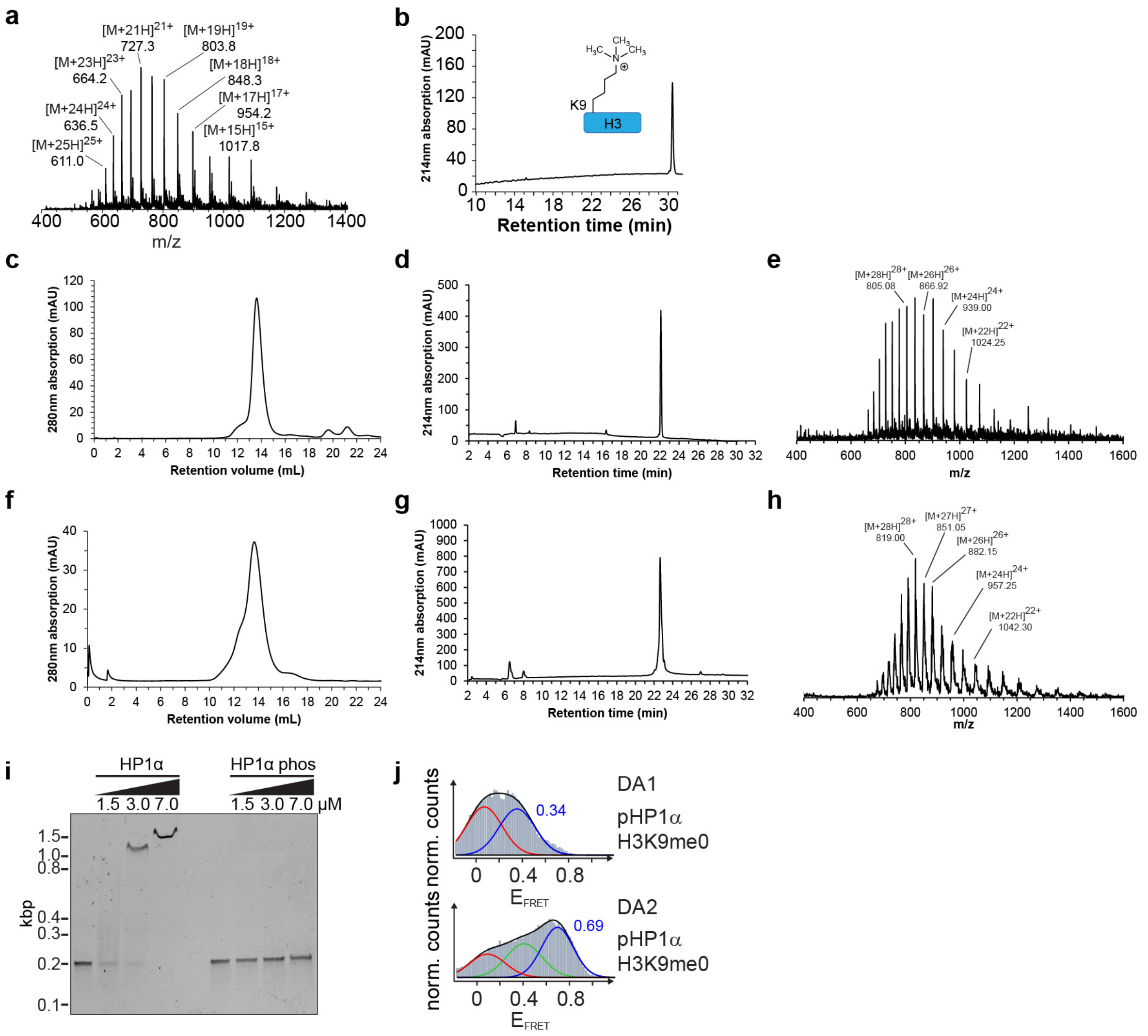

Supplementary Figure 19 | Expression and purification of wt and phosphorylated HP1α. H3K9me3 is produced by expressed protein ligation. The modified histone peptide H3(1-14)K9me3-$NHNH_2$ is converted to a C-terminal thioester and ligated to the N-terminally truncated histone protein H3(Δ1-14)A15C, followed by desulfurization[8]. (**a**) MS spectrum of semisynthetic H3K9me3. (Expected mass: 15251.8Da, observed mass 15252.0 Da) (**b**) RP-HPLC analysis of H3K9me3. (**c**) Gel-filtration analysis of HP1α, which elutes at the expected volume for the dimeric protein. (**d**) HPLC analysis of HP1α with a 0-70%B gradient. (**e**) ESI-MS analysis of HP1α (Expected mass 22506.2Da, observed mass 22513.0Da) (**f**) Gel-filtration analysis of phosphorylated HP1α (phosHP1α), which elutes at the expected volume for the dimeric protein with a shoulder potentially accounting for a tetrameric population. (**g**) HPLC analysis of phosHP1α with a 0-70%B gradient. (**h**) ESI-MS phosHP1α (Expected mass 22906.2Da, observed mass 22905.0), demonstrating the presence of 5 P_i groups. (**i**) Gel-shift with HP1α and phosHP1α demonstrating a loss in nonspecific DNA binding affinity for the phosphorylated protein in accordance with ref. [9]. (**j**) Histograms of pHP1a incubated with DA1 or DA2 containing unmethylated H3.

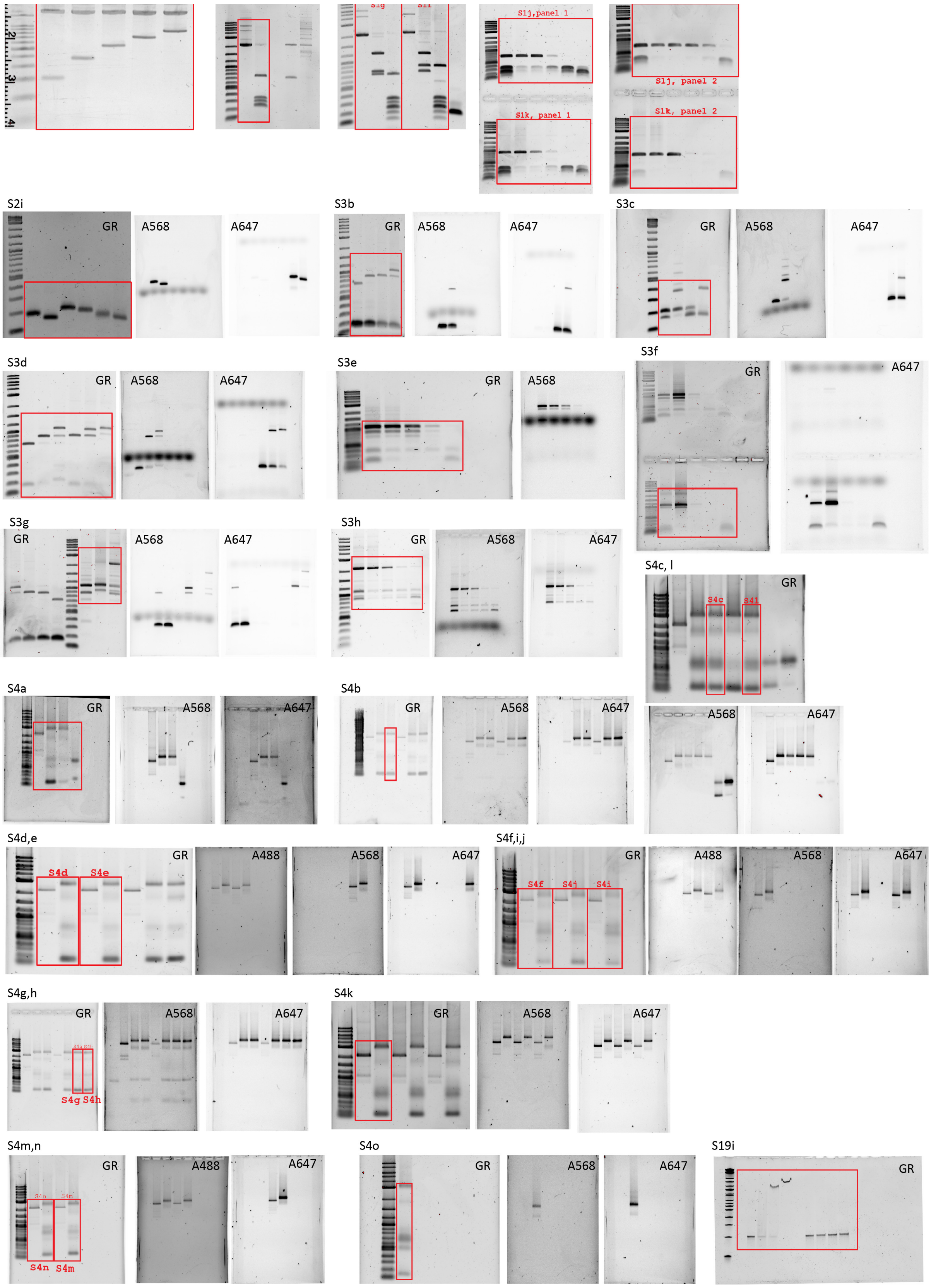

Supplementary Figure 20 | Images of all uncropped gels. All uncropped gels from Supplementary Figures S1, S2, S3, S4 and S19, stained with GelRed (GR) or imaged using fluorescence from the indicated dyes.

Supplementary Tables

Fragment	Sequence
P1	-100 -95 -90 -85 -80 -75 -70 -65 -60 -55 -50 -45 -40 -35 -30 -25<br>CACTTGGTGGCGGCCGCC**CTGGAGAATCCCGGTGCCGAGGCCGCTCAATTGGTCGTAGACAGCTCTA**<br>-20 -15 -10 -5 0 5 10 15 20 25 30 35 40 45 50 55<br>**GCACCGCTTAAACGCACGTACGCGCTGTCCCCCGCGTTTTAACCGCCAAGGGGATTACTCCCTAGTCTCCAGGCACGTGT**<br>60 65 70 75 80 85 90 95 100 105<br>**CAGATACTGCAGAGATCT**CTAGATCCATGGAGTACTTGGTCTCATAGC
P2	-100 -95 -90 -85 -80 -75 -70 -65 -60 -55 -50 -45 -40 -35 -30 -25<br>GATCGGTCTCATAGC**CTGGAGAATCCCGGTGCCGAGGCCGCTCAATTGGTCGTAGACAGCTCTA**<br>-20 -15 -10 -5 0 5 10 15 20 25 30 35 40 45 50 55<br>**GCACCGCTTAAACGCACGTACGCGCTGTCCCCCGCGTTTTAACCGCCAAGGGGATTACTCCCTAGTCTCCAGGCACGTGT**<br>60 65 70 75 80 85 90 95 100 105<br>**CAGATATATACATCCTGT**CACACTGTGGATC
P3	-100 -95 -90 -85 -80 -75 -70 -65 -60 -55 -50 -45 -40 -35 -30 -25<br>CACACTGTGCCAAGTACT**T**ACGCGGCCGCC**CTGGAGAATCCCGGTGCCGAGGCCGCTCAATTGGTCGTAGACAGCTCTA**<br>-20 -15 -10 -5 0 5 10 15 20 25 30 35 40 45 50 55<br>**GCACCGCTTAAACGCACGTACGCGCTGTCCCCCGCGTTTTAACCGCCAAGGGGATTACTCCCTAGTCTCCAGGCACGTGT**<br>60 65 70 75 80 85 90 95 100 105<br>**CAGATACTGCAGAGATCT**CTAGATCC**A**TGGAGTACTTGGTCTCAAACC
P4	-100 -95 -90 -85 -80 -75 -70 -65 -60 -55 -50 -45 -40 -35 -30 -25<br>GATCGGTCTCAAACC**CTGGAGAATCCCGGTGCCGAGGCCGCTCAATTGGTCGTAGACAGCTCTA**<br>-20 -15 -10 -5 0 5 10 15 20 25 30 35 40 45 50 55<br>**GCACCGCTTAAACGCACGTACGCGCTGTCCCCCGCGTTTTAACCGCCAAGGGGATTACTCCCTAGTCTCCAGGCACGTGT**<br>60 65 70 75 80 85 90 95 100 105<br>**CAGATATATACATCCTGT**CACGTCGTGGATC
P5	-100 -95 -90 -85 -80 -75 -70 -65 -60 -55 -50 -45 -40 -35 -30 -25<br>CACGTCGTGCCAAGTACTTACGCGGCCGCC**CTGGAGAATCCCGGTGCCGAGGCCGCTCAATTGGTCGTAGACAGCTCTA**<br>-20 -15 -10 -5 0 5 10 15 20 25 30 35 40 45 50 55<br>**GCACCGCTTAAACGCACGTACGCGCTGTCCCCCGCGTTTTAACCGCCAAGGGGATTACTCCCTAGTCTCCAGGCACGTG**<br>60 65 70 75 80 85 90 95 100 105<br>**TCAGATACTGCAGAGATCT**CTAGATCCGGTCTCACTAA

Supplementary Table 1 \| Sequences of 1x601 pieces for recombinant and PCR-generated pieces. 601 sequences (for recombinant pieces with slight end modifications) indicated in bold. The labeled base pairs are indicated in red. The numbering is given as number of base-pairs relative to the dyad in the 601 sequence.

Name	Sequence
P2_pos39_rev	5'-GATCCACAGTGTGACAGGATGTATATATCTGACACGTGCCTGGAGACTAGGGAG-3'
P2_pos71_rev	5'-GATCCACAGTGTGACTGGATGTATATATCTGACACGTGC-3'
P3_pos-86_fwd	5'-GATCGCACACTGTGCCAAGTACTTACGCGGCCGCCCTGGAGAATCC-3'
P3_pos82_rev	5'-GATCGCGGTTTGAGACCAAGTACTCCATGGATCTAGAGATCTCTGC-3'
P4_pos-39_fwd	5'-GATCGGTCTCAAACCCTGGAGAATCCCGGTGCCGAGGCCGCTCAATTGGTCGTAGACAGC3'
P4_pos-16_fwd	5'-GATCGGTCTCAAACCCTGGAGAATCCCGGTGCCGAGGCCGCTCAATTGGTCGTAGA-<br>CAGCTCTAGCACCGCTTAAACGCACGTACGC-3'
recP5_Anchor_fwd	5'-ph-CTAATAGTCTGCTCAGTACTCGTCGCTAGATCCATGGTCCGATTACGCGG-3'
recP5_ Anchor _rev	5'-biotin-CCGCGTAATCGGACCATGGATCTAGCGACGAGTACTGAGCAGACTA-3'

Supplementary Table 2 | Oligonucleotides sequences used for labeling and anchoring. The numbering for the label positions is given relative to the nucleosome dyad.

Piece	Donor fluorophore	Labeling position(s)	Acceptor fluorophore	Labeling position(s)
P2	Alexa Fluor 488	39	-	-
P2	Alexa Fluor 568	39	-	-
P2	Alexa Fluor 488	71	-	-
P2	Alexa Fluor 568	71	-	-
P2	-	-	-	-
P3	Alexa Fluor 488	-86	Alexa Fluor 647	82
P3	Alexa Fluor 568	-86	Alexa Fluor 647	82
P3	-	-	-	-
P4	-	-	Alexa Fluor 647	-39
P4	-	-	Alexa Fluor 647	-16
P4	-	-	-	-

Supplementary Table 3 | Overview of 1x601 PCR pieces generated from labeled and unlabeled primers. Position of label and the donor/acceptor fluorophore used are indicated. The positions are indicated as base-pairs relative to the dyad in the 601 sequence (see also **Supplementary Tables 1** and **2**).

Construct	Donor	Donor Position	Acceptor	Acceptor position
DA1	Alexa488	N5 (39)	Alexa647	N7 (-39)
DA1	Alexa568	N5 (39)	Alexa647	N7 (-39)
D1	Alexa488	N5 (39)	unlabeled	N/A
DA1’	Alexa488	N5 (39)	Alexa647	N6 (-39)
DA2	Alexa488	N5 (71)	Alexa647	N7 (-16)
DA2	Alexa568	N5 (71)	Alexa647	N7 (-16)
D2	Alexa488	N5 (71)	unlabeled	N/A
DA3	Alexa488	N6 (-86)	Alexa647	N6 (82)
DA3	Alexa568	N6 (-86)	Alexa647	N6 (82)
D3	Alexa488	N6 (-86)	unlabeled	N/A

Supplementary Table 4 | Overview of constructed 12x601 DNA pieces with different combinations of labels. The nucleosome position is indicated as N*i*, with *i* = 1-12. The number in brackets is the label position relative to the dyad in the 601 sequence (see also **Supplementary Tables 1**-**3**).

Data		n	Peak 1			Peak 2			Peak 3		
			A_1	C_1	σ_1	A_2	C_2	σ_2	A_1	C_1	σ_1
DA1	0mM Mg^{2+}	448	2.45	0.07	0.17	5.02	0.30	0.14	-		-
	0.5mM Mg^{2+}	205	1.81	0.09	0.17	5.18	0.36	0.14	-	-	-
	1.0mM Mg^{2+}	156	1.68	0.12	0.22	5.13	0.45	0.14	-	-	-
	4.0mM Mg^{2+}	789	1.65	0.07	0.22	4.17	0.58	0.17	-	-	-
	4.0mM Mg^{2+} Ac	146	3.76	0.08	0.20	2.38	0.40	0.17	-	-	-
	HP1a K9me0	964	1.88	0.01	0.14	4.02	0.30	0.20	-	-	-
	HP1a K9me3	797	1.79	0.04	0.17	3.82	0.38	0.20	-	-	-
	HP1a_p K9me0	179	3.32	0.04	0.14	3.19	0.33	0.17	-	-	-
	HP1a_p K9me3	262	1.28	0.03	0.17	3.88	0.44	0.22	-	-	-
DA2	0mM Mg^{2+}	174	5.37	0.00	0.14	0.44	0.90	0.10	0.92	0.57	0.22
	0.5mM Mg^{2+}	132	3.98	0.01	0.14	1.88	0.65	0.22	0.81	0.27	0.10
	1.0mM Mg^{2+}	103	2.83	0.02	0.17	2.07	0.75	0.17	1.23	0.45	0.22
	4.0mM Mg^{2+}	138	2.86	0.00	0.14	1.96	0.70	0.22	1.31	0.30	0.22
	4.0mM Mg^{2+} Ac	113	1.35	0.01	0.22	2.13	0.62	0.20	1.84	0.25	0.20
	HP1a K9me0	260	2.45	0.01	0.17	1.52	0.61	0.22	1.81	0.30	0.17
	HP1a K9me3	252	1.20	0.05	0.22	3.49	0.71	0.17	1.12	0.41	0.17
	HP1a_p K9me0	901	0.63	0.04	0.17	3.01	0.66	0.17	2.21	0.33	0.22
	HP1a_p K9me3	275	1.67	0.07	0.22	2.40	0.74	0.17	1.37	0.36	0.22
DA3	0mM Mg^{2+}	216	1.40	0.03	0.17	5.90	0.74	0.10	1.34	0.58	0.22
	0.5mM Mg^{2+}	165	0.85	0.05	0.20	7.57	0.78	0.00	2.94	0.72	0.14
	1.0mM Mg^{2+}	155	1.29	0.03	0.14	6.08	0.81	0.10	2.66	0.75	0.17
	4.0mM Mg^{2+}	145	1.34	0.02	0.14	6.75	0.87	0.00	3.39	0.79	0.14
	4.0mM Mg^{2+} Ac	130	0.45	0.04	0.22	7.13	0.86	0.10	2.14	0.70	0.22

Supplementary Table 5 | Gaussian fits of FRET distributions from Fig. 2 and 4. Histograms of the E_{FRET} values of combined traces (number of traces: *n*) were fitted using the indicated number of Gaussians with amplitude A_i, center c_i and width σ_i. Ac: Chromatin fiber contains $H4K_S16Ac$

	0mM Mg^{2+}	4.0mM Mg^{2+}	4.0mM Mg^{2+} Ac	HP1	
				H3K9me0	H3K9me3
**DA1**	17 %	29 %	22 %	55 %	54 %
**DA2**	35 %	38 %	26 %	38 %	42 %
**DA3**	20 %	20 %	19 %	X	X

Supplementary Table 6 | Percentage of dynamic traces observed in smTIRF. Traces are identified as dynamic by cross-correlation (CC) analysis of donor versus acceptor fluorescence emission. Dynamic traces exhibit a CC amplitude of < -0.1 and a CC relaxation time > 100 ms.

Contributions to **ΔR_{DA}**	**DA1**	**DA2**	**DA3**
Calibration contributions to the uncertainty, Δ$R_{DA,cal}$ in **step 2** [a]			
Uncertainty of the correction factor, γ, ΔR_{DA} ($\Delta\gamma$), mainly due to $\Delta\Phi_{FA}$ (Summary 9.3: $\langle\Phi_{FA}\rangle$ = 0.368 with $\Delta\Phi_{FA}$ = 0.017)	0.008	0.008	0.008
Uncertainty of correction factors, α, β and δ, ΔR_{DA}($\Delta\alpha$,$\Delta\beta$,$\Delta\delta$)	< 0.005	< 0.005	< 0.005
Uncertainty of signal correction by background, $B_{Xem\|Xex}$, $\Delta B_{Xem\|Xex}$	< 0.005	< 0.005	< 0.005
Contributions to the uncertainty ΔR_0 [b]			
Uncertainty of refractive index, $\Delta R_0(n)$ [c]	0.040	0.040	0.040
Uncertainty of donor fluorescence quantum yield Φ_{FD}, $\Delta R_0(\Phi_{FD})$ [c]	0.020	0.020	0.020
Uncertainty of spectral overlap integral J, $\Delta R_0(J)$ [c]	0.025	0.025	0.025
Uncertainty of FRET orientations factor, κ^2, $\Delta R_0(\kappa^2)$ [d]	0.071	0.070	0.060
**Total accuracy ΔR_0**	**0.088**	**0.087**	**0.079**
Noise contributions to the uncertainty Δ$R_{DA,noise}$(R_{DA})			
Precision to fit R_{DA} by dynPDA, ΔR_{DA}(R_{DA}) [e]	0.020	0.030	0.020
Uncertainty by A heterogeneity, ΔR_{DA}(A_{het}) [f]	0.0001	0.0038	0.0051
**Total uncertainty** to compute $R_{DA,}$ ΔR_{DA}(Δ$R_{DA,cal}$, ΔR_0, Δ$R_{DA,noise}$(R_{DA}, ΔA_{het}))[g]	**0.090**	**0.092**	**0.081**

[a] Adapted from ref. [10].
[b] Adapted from ref. [11].
[c] Values from ref. [11].
[d] We computed the densities of κ^2 (see section 3.5 of ref. [12]) using the residual anisotropies of the donor, $r_{inf,D}$ (Summary 9.2), and of the acceptor, $r_{inf,A}$ (Summary 9.4), to determine the uncertainty $\Delta\kappa^2$ and the corresponding $\Delta R_0(\kappa^2)$.
[e] See Step 9, section **Determination of uncertainties in measured R_{DA} distances** and **Supplementary Figures 16-18**.
[f] See step 9, Summary 9.3.
[g] Calculated according to eq. (9.7).

Supplementary Table 7 | Accuracy and precision of the inter-dye distance calculation R_{DA}. Relative uncertainties are reported for individual contributions (see **Supplementary Note, Step 9**) followed by the calculated total accuracy ΔR_0, and the total uncertainty ΔR_{DA}. For values in % multiply by 100. Error propagation was performed according to refs. [10,11] using eq. (9.7).

	Model	Experiment	Model	Experiment
	$\langle R_{DA,m}\rangle_E$, register 1, (Å)	$\langle R_{DA,exp}\rangle_E$, register 1, (Å)	$\langle R_{DA,m}\rangle_E$, register 2, (Å)	$\langle R_{DA,exp}\rangle_E$, register 2, (Å)
**DA1**	64	64	47	46
**DA2**	41	53	36	44
**DA3**	48 [a]	42-48	53 [a]	42-48

[a] linear linker DNA to connect the two nucleosomes in a register was assumed.

Supplementary Table 8 | Correlation between FRET-averaged inter-dye distances of the structural models, $\langle R_{DA,m}\rangle_E$, and of the experiments, $\langle R_{DA,exp}\rangle_E$, for the Alexa488/647 FRET pair. Experimental distances $\langle R_{DA,exp}\rangle_E$ of DA 1-3 were obtained from PDA analysis (**Supplementary Figures 16 - 18**). The model distances were calculated for the tetranucleosome model (register 1 and 2) considering the total experimental uncertainty $\Delta R_{DA}(\Delta E_{FRET}, \Delta R_0, \Delta R_{DA,noise}(R_{DA}, \Delta A_{het}))$ (**Supplementary Table 7**) using ACV analysis (**Supplementary Note, step 9,** sections Determination of the uncertainties for structural modeling (ACV parameters), and FRET positioning and screening calculations (**Supplementary Figures 12 and 13**). Considering DA2, there are two possible explanations for the deviations between $\langle R_{DA,m}\rangle_E$ and $\langle R_{DA,exp}\rangle_E$: (1) Local dynamics could be present (clamshell and torsion by 10 degrees, see **Supplementary Fig. 13**). (2) In addition, in view of the low Mg^{2+} concentrations and the absence of H1, the stacking of the nucleosome arrays could differ in solution from that in the X-ray[13] or cryoEM[5] structure.

Supplementary Note

Dynamic structural biology analysis: 30
Step 1. smTIRF 32
Step 2: 2D MFD plots with FRET lines and calibration of the FRET measurements 33
Calculation of FRET efficiencies EFRET from fluorescence signals 34
Expanding the dynamic range of smFRET studies. 35
Static and dynamic FRET lines. 35
Step 3: Sub-ensemble TCSPC 36
Step 4: Burst-ID FCS 38
Step 5: Photobleaching and photoblinking analysis 39
Step 6. Evaluation of kinetic networks between FRET species compatible with experimental data 40
Step 7. General description of PDA analysis 40
Calculation of donor acceptor distances from fluorescence signals 41
Dynamic PDA. 41
Step 8: Validation of kinetic models. 43
Step 9: Relating measured R_{DA} distances to structural models of compact chromatin states 43
Determination of the uncertainties for structural modeling. 44
Summary of dye properties of the donor Alexa488. 44
Summary of acceptor dye properties. 45
Determination of uncertainties in measured R_{DA} distances 47
Model building. 48
FRET positioning and screening calculations. 48
Step 10: Structural models of open and dynamic states 49
Coarse grained simulations 50
Step 11: Final model and its validation - A unified model of chromatin dynamics 51

Dynamic structural biology analysis:

We used a combination of experimental observables (described in detail below) for structural and kinetic analyses to establish a model for chromatin dynamics, as shown in **Fig. 6**. We established an 11-step workflow for dynamic structural biology (**Supplementary Fig. 9**), involving a sequence of steps:

Step 1: Measuring FRET efficiencies over time in **smTIRF** we explored chromatin dynamics in the 100 ms – seconds regime. Employing cross-correlation analysis we observed that between 20-55% of fibers showed dynamics on the 50-500 ms timescale (**Fig. 2** and **Supplementary Table 6**).

Step 2: 2D MFD plots. We measured smFRET with confocal multi-parameter fluorescence detection (MFD). E_{FRET} vs fluorescence-weighted average fluorescence lifetime of the donor dye in presence of the acceptor $\langle \tau_{D(A)} \rangle_F$ enabled us to identify rapid dynamics and allowed us to detect the coexisting dynamic chromatin populations in two different tetranucleosome interaction registers (register 1 and 2). In the presence of dynamic exchange between conformations with different E_{FRET} and exchange kinetics faster than the molecular dwell time in the confocal detection volume (< 10 ms), deviations from the ideal relationship between $\langle \tau_{D(A)} \rangle_F$ and E_{FRET} (the static FRET line) can be detected in burst-wise analysis[14]. This is because E_{FRET} values derived from fluorescence intensities are averaged per molecular species fractions, whereas average fluorescence lifetimes are computed per brightness by the applied maximum-likelihood analysis[15]. This disagreement is captured by a dynamic FRET line. For the chromatin arrays DA1-3 MFD plots indeed directly indicated a dynamic process as a large fraction of the detected molecules fell on dynamic FRET-lines (**Fig. 3d,e**). Importantly, measurements with DA1, DA2 or DA3 labeled DNA (absence of histones) as well as measurements with chromatin samples bearing only donor dyes (D1, D2 and D3; **Supplementary Table 4**) did not show comparable FRET states or dynamics (**Supplementary Fig. 8c,d**).

Step 3: Sub-ensemble Time Correlated Single Photon Counting (seTCSPC) resolved the FRET efficiency levels corresponding to chromatin structural states (**Supplementary Fig. 10**).

Step 4: Burst-ID fluorescence correlation spectroscopy (FCS) of donor-donor, donor-acceptor or cross-correlation confirmed complex sub-millisecond dynamics in a model-free approach and yielded estimates of the involved timescales (DA1-3, 0.75 mM Mg^{2+}, **Supplementary Fig. 11**).

Step 5: Photobleaching and photoblinking analysis was employed to confirm that the observed dynamic processes originate from structural transitions and did not contain contributions from photophysics of the dyes (**Supplementary Fig. 14a**).

Steps 6-11: The combined obtained data was used to **formulate kinetic models and assign states and connectivity between the states** (**step 6**). Subsequently a unified kinetic model was used in dynamic PDA analysis (**step 7, Fig. 5** and **Supplementary Fig. 16-18**). A global fit to the experimental data yields improved FRET efficiency levels values with corresponding population fractions and exchange rate constants. Afterwards the model was judged by applying a selection of criteria (**Supplementary Fig. 15**) including an evaluation of goodness of fit, the stability of the fit results (**step 8**) as well as by determination of the parameter uncertainties (**step 9**) and structural validation such as atomistic models (**step 9**) and coarse grained simulations (**step 10**). The procedure finally results in a complete model of chromatin dynamics (**step 11**).

Step 1. smTIRF

From donor- (F_D) and acceptor fluorescence emission intensity (F_A) traces FRET efficiency (E_{FRET}) traces were calculated, using

$$E_{FRET} = \frac{F_A - \beta F_D}{F_A - \beta F_D + \gamma F_D} \text{ and } \gamma = \frac{\Delta F_{A,bleach}}{\Delta F_{D,bleach}} \quad (0.1)$$

The values of $\beta = 0.141$ and $\gamma = 0.468$ were experimentally determined for the dye pair Alexa568/647 in our experimental setup. The bin size for all histograms was set to 0.02. E_{FRET} histograms of each trace of length > 5 s were normalized to total counts. Final histograms were calculated averaging the FRET histograms of all traces (> 100 traces) and fitted using 2 or 3 Gaussian functions $\sum_i A_i e^{-(x-c_i)^2/\sigma_i}$ (**Supplementary Table 5**).

Cross-correlation analysis was performed using

$$C_{cross}(t) = \langle \Delta F_D(0) \cdot \Delta F_A(t) \rangle / \langle \Delta F_D(0) \cdot \Delta F_A(0) \rangle \quad (0.2)$$

where ΔF_D and ΔF_A are the variances of donor and acceptor fluorescence at time 0 or t, was calculated in Matlab using a maximum lag of 10 s. Traces shorter than 10 s, as well as traces which spent less than 20% of the time at $E_{FRET} < 0.2$ were excluded from the analysis. The cross-correlation data was fitted with a bi-exponential function in OriginPro (OriginLab Corporation). To determine the fraction of dynamic traces (**Supplementary Table 6**) the individual cross-correlation decays from each trace was analyzed. Traces considered dynamic showed an amplitude < -0.1 and a decay time constant > 0.1 s.

This analysis revealed that chromatin fibers exhibit dynamics on the 50-500 ms timescale, but that such fluctuations were only observed in a subset of individual arrays. This argues for the existence of 'locked' states where tetranucleosome interactions are stable over time (**Figure 6**).

Step 2: 2D MFD plots with FRET lines and calibration of the FRET measurements

Burst selection. The bursts of all samples were identified and selected from the MFD data trace as described in ref. [16]. Double-labeled chromatin arrays with the DA pair Alexa488/647 capable for FRET (FRET-active) were selected by Pulsed Interleaved Excitation (PIE) using E_{FRET} vs S (stoichiometry) 2D histograms. For subsequent analysis we selected bursts with $0.2 < S < 0.8$ to separate double-labeled species from single dye labeled molecules and $|T_{GX}\text{-}T_{RR}| < 1$ ms to remove contributions from photophysical processes [17] (**Supplementary Fig. 8a**). For PIE[18], the corrected stoichiometry S is defined as

$$S = \frac{F_{D|D} + F_{A|D}}{F_{D|D} + F_{A|D} + F_{A|A}} \tag{2.1}$$

$F_{Xem|Xex}$ corresponds to a fully corrected fluorescence intensity computed from observed signal ${}^{obs}S$ considering background intensities and other experimental correction factors α, β, γ, δ defined in eq. (2.3). The meaning of the indices is as follows: (D/D) is the donor intensity if the donor was excited, (A/D) is the acceptor intensity if the donor was excited and (A/A) is the acceptor intensity if the acceptor was excited.

The stoichiometry S is computed from the observed signals ${}^{obs}S$ in two steps:

(i) The registered primary signal ${}^{obs}S$ was corrected for the mean background $\langle B \rangle$ signal contribution in the green and red channels, respectively:

$$I_{Xem|Xex} = {}^{obs}S_{Xem|Xex} - \langle B_{Xem|Xex} \rangle \tag{2.2}$$

$I_{Xem|Xex}$ corresponds to a background corrected signal: $I_{G|G}$ is the background corrected signal in the donor channel (G) after donor excitation (G), $I_{R|G}$ is the background corrected signal in the acceptor channel (R) at wavelength G for donor excitation and $I_{R|R}$ is the background corrected signal in the acceptor chanel after acceptor excitation, respectively.

(ii) The background corrected signals I were used together with four correction factors α, β, γ, δ to compute S according to:

$$S = \frac{\gamma \cdot I_{G|G} + \left(I_{R|G} - \left(\alpha \cdot I_{G|G} + \beta \cdot I_{R|R}\right)\right)}{\gamma \cdot I_{G|G} + \left(I_{R|G} - \left(\alpha \cdot I_{G|G} + \beta \cdot I_{R|R}\right)\right) + \frac{1}{\delta} \cdot I_{R|R}} \tag{2.3}$$

with $\alpha = \frac{g_{R|D}}{g_{G|D}}$; $\beta = \frac{\sigma_{A|G} L_G}{\sigma_{A|R} L_R}$; $\gamma = \frac{g_{R|A}\ a\ \Phi_{F,A}}{g_{G|D} \Phi_{F,D(0)}}$; $\delta = \frac{\sigma_{A|R}}{\sigma_{D|G}} \frac{L_R}{L_G}$

The parameter α is a correction factor for the spectral donor fluorescence crosstalk (leakage) into the red "acceptor" detection channel. β normalizes the direct acceptor excitation rates in the FRET experiment to that in the PIE experiment defined by the acceptor excitation cross-sections $\sigma_{A|G}$ at donor excitation and

$\sigma_{A|R}$, and the direct excitation irradiances [Photons/cm^2] L_G and L_R for the donor and acceptor at the wavelengths G and R. γ is a correction factor for the fluorescence quantum yields $\Phi_{F,D(0)}$, $\Phi_{F,A}$, the fraction of the fluorescent trans state of Alexa647 *a*, and the detection efficiencies of the green donor $g_{G|D}$ and the red acceptor channel $g_{R|A}$, respectively. δ normalizes the donor excitation rate for the FRET studies to the direct acceptor excitation rate of the PIE experiment defined by the excitation cross-sections for D $\sigma_{D|G}$ and A $\sigma_{A|R}$ respectively, and the direct excitation irradiances [photons/cm^2] L_G and L_R for the donor and acceptor at the wavelengths *G* and *R*.

For chromatin samples with the FRET pair Alexa568/647 we did not employ PIE (i.e. δ is n.a.). The direct acceptor excitation rate at the donor excitation wavelength (β >> 0) could however be used to identify double-labeled DA species. Thus, double-labeled chromatin arrays with the DA pair Alexa568/647 capable for FRET (FRET-active) were selected by a minimal number of acceptor photons (red cut) due to direct acceptor excitation by L_G. In this way contributions from DOnly molecules were reduced. The following parameters were used for the two studied FRET pairs for given experimental setup (see **MFD measurement procedures**):

**parameter**	**FRET pair D/A**	
	**Alexa488/647**	**Alexa568/647**
α	0.016	0.146
β	0	0.131
$g_{G\|D}/g_{R\|A}$	0.95	1.45
$\Phi_{F,D(0)}$ [a]	0.8	0.69
a $\Phi_{F,A}$ [a]	0.368	0.368
γ	0.5	0.38
δ	0.83	n.a.

[a] average values for the FRET pairs DA1-3. The determined values are compiled in the summary tables reported in **step 9**.

Calculation of FRET efficiencies EFRET from fluorescence signals

The corrected FRET efficiency E_{FRET} is defined via fully corrected fluorescence intensities *F*:

$$E_{FRET} = \frac{F_{A|D}}{F_{D|D} + F_{A|D}} \tag{2.4}$$

In analogy to *S*, E_{FRET} can be computed by the observed intensities and corresponding correction parameters α, β, γ defined in eq. (2.3):

$$E_{FRET} = \frac{\left(I_{R|G} - \left(\alpha \cdot I_{G|G} + \beta \cdot I_{R|R}\right)\right)}{\gamma \cdot I_{G|G} + \left(I_{R|G} - (\alpha \cdot I_{G|G} + \beta \cdot I_{R|R})\right)} \tag{2.5}$$

Expanding the dynamic range of smFRET studies. We used the FRET pairs Alexa568/647 (Förster Radius R_0 = 82 Å) and Alexa488/647 (R_0 = 52 Å) to exploit different distance sensitivities (**Fig. 3d,e**): Alexa568/647 allows for the detection of long-range dynamics beyond 120 Å, whereas Alexa488/647 enables the investigation of sub-states and their exchange dynamics below 60 Å.

Static and dynamic FRET lines. All MFD plots (**Fig. 3d,e**) for DA1-3 (Alexa488/647 and Alexa568/647) are presented with static and dynamic FRET lines, to demonstrate the presence of two distinct chromatin populations (register 1 and 2). Each population exhibits kinetic exchange faster than the molecular dwell time (< 10 ms) within the bursts. The theoretical dependence between FRET efficiency and species weighted average donor fluorescence lifetime in presence of acceptor dye is described as

$$E_{static} = 1 - \frac{\langle \tau \rangle_x}{\tau_{D(0)}}. \quad (2.6)$$

Here we use an empirical dependence of species weighted average donor lifetime $\langle\tau\rangle_x$ on fluorescence weighted average donor lifetime $\langle\tau\rangle_F$ as a polynomial with c_i coefficients obtained by numerical simulations[12]

$$\langle \tau \rangle_x = \sum_{i=0}^{n} c_i \left(\langle \tau \rangle_F\right)^i. \quad (2.7)$$

Here we used the following joint parameters for DA1-3 constructs, which are common for the two FRET pairs Alexa568/647 and Alexa568/647, respectively:

Alexa568/647 labeling (**Fig. 3d**): c_0=-0.0083, c_1=0.0848, c_2=0.2926, c_3=-0.6606, c_4=0.0085 with $\tau_{D(0)}$=3.5 ns.

and

Alexa488/647 labelling (**Fig. 3e**) c_0=-0.0056, c_1=0.0838, c_2=0.4007, c_3=-0.3806, c_4=0.00225 with $\tau_{D(0)}$=4.0 ns.

The dynamic FRET line are described as

$$E_{dyn} = 1 - \frac{\tau_{F1} \cdot \tau_{F2}}{\tau_{D(0)} \cdot \left(\tau_{F1} + \tau_{F2} - \langle \tau \rangle_x\right)} \quad (2.8)$$

where τ_{F1} and τ_{F2} are the donor fluorescence lifetimes defining the limiting FRET states of the respective line. We have assumed that the limiting states of each DA sample remain the same for all Mg^{2+} concentrations.

<u>Alexa568/647, dynamic FRET line for register 1</u> between A and D states with $\tau_{D(0)}$=3.5 ns (**Fig. 3d**, dark blue):

DA1 between A/C and D states: τ_{F1} =0.8 ns, τ_{F2} =3.45 ns, c_1=1.2458, c_2=0.84821.

DA2 between A and C: τ_{F1} =0.56 ns, τ_{F2} =1.2 ns, c_1=1.4285, c_2=-0.5695; and between C and D: τ_{F1} =1.15 ns, τ_{F2} =3.5 ns, c_1=1.1778, c_2=0.6221.

DA3 between A/B and C: τ_{F1}=0.25 ns, τ_{F2} =0.8 ns, c_1=1.626, c_2=0.5523; and between C and D: τ_{F1}=0.8 ns, τ_{F2} =3.5 ns, c_1=1.2473, c_2=0.8655.

Alexa568/647, dynamic FRET line for register 2 between B and D states with $\tau_{D(0)}$=3.5 ns (**Fig. 3d**, light blue):

DA1-3: τ_{F1}=0.25 ns, τ_{F2} =3.5 ns, c_1=1.5198, c_2=1.819.

Alexa488/647, dynamic FRET line for register 1 between A and D states with $\tau_{D(0)}$=4.0 ns (**Fig. 3e**, dark blue):

DA1: τ_{F1}=3.12 ns, τ_{F2} =3.92 ns, c_1=1.0895, c_2=0.317;

DA2: τ_{F1}=2.53 ns, τ_{F2} =3.96 ns , c_1=1.1605, c_2=0.6339;

DA3: τ_{F1}=2.57 ns, τ_{F2} =3.96 ns, c_1=1.154, c_2=0.6082.

Alexa488/647, dynamic FRET line for register 2 between B and D states with $\tau_{D(0)}$=4.0 ns (**Fig. 3e**, light blue):

DA1 and DA2: τ_{F1}=1.55 ns, τ_{F2} =3.92 ns, c_1=1.376, c_2=-1.4852;

DA3: τ_{F1}=1.6 ns, τ_{F2} =3.95 ns, c_1=1.3607, c_2=-1.4248.

Importantly, measurements with DA1, DA2 or DA3 double labeled DNA (absence of histones) as well as measurements with chromatin samples bearing only donor dyes (D1, D2 and D3; **Supplementary Table 4**) did not show comparable FRET states or dynamics (**Supplementary Fig. 8c,d**).

Step 3: Sub-ensemble TCSPC

For defining the limiting states for dynamic FRET lines indicated by FRET efficiency levels (**Fig. 3e** orange, wine and gray lines), we performed sub-ensemble Time Correlated Photon Counting (seTCSPC) analysis of DA1-3 (Alexa488/647), which were selected from the sample as double-labeled species by PIE (**Supplementary Fig. 8a**). Characteristic populations for each respective limiting state are analyzed for bursts with a low FRET efficiencies (0< E_{FRET} <0.199) of the low FRET population (LF) and for bursts with higher FRET efficiencies (0.2< E_{FRET} <1.0) of the dynamic FRET population (dynF). To retrieve the required information about the limiting states, we analyzed bursts of each population separately. The specific fluorescence decays were analyzed by a fit model described previously[19]. The fluorescence decay of the donor reference (DOnly, in the absence of FRET) was approximated by a single fluorescence lifetime, $\tau_{D(0)}$:

$$f_{D(0)}(t) = f_{D(0)}(0)\exp(-t/\tau_{D(0)}) \quad (3.1)$$

Hence, the FRET-rate (k_{FRET}) is only determined by the donor-acceptor distance and their relative orientation. In the presence of FRET, the donor fluorescence decay can be expressed using the donor-acceptor distance distribution $p(R_{DA})$:

$$f_{\mathrm{D(A)}}(t) = f_{\mathrm{D(A)}}(0) \int_{R_{DA}} p(R_{\mathrm{DA}}) \exp\left(-\frac{t}{\tau_{\mathrm{D(0)}}} \left[1 + (R_0 / R_{\mathrm{DA}})^6 \right] \right) dR_{\mathrm{DA}} \tag{3.2}$$

Here we assumed Gaussian distribution of donor-acceptor distances ($p(R_{DA})$) with a mean of $\langle R_{DA} \rangle$ and a half-width of σ_{DA} which is expressed as:

$$p(R_{\mathrm{DA}}) = \frac{1}{\sqrt{2\pi}\sigma_{\mathrm{DA}}} \exp\left(-\frac{(R_{\mathrm{DA}} - \langle R_{\mathrm{DA}} \rangle_{\exp})^2}{2\sigma^2_{\mathrm{DA,exp}}} \right) \tag{3.3}$$

In addition, a fraction of Donor-only molecules (x_{DOnly}) and a constant offset c was considered to describe the experimentally observed fluorescence decay $f(t)$:

$$f(t) = (1 - x_{\mathrm{DOnly}}) \cdot f_{\mathrm{D(A)}}(t) + x_{\mathrm{DOnly}} \cdot f_{\mathrm{D(0)}}(t) + c \tag{3.4}$$

Combining the donor fluorescence decay in the presence, $f_{D(A)}(t)$, and in the absence, $f_{D(0)}(t)$, of FRET by a time-dependent ratio a measure of FRET, $\varepsilon_D(t)$, is obtained:

$$\varepsilon_D(t) = \frac{f_{D(A)}(t)}{f_{D(0)}(t)} \tag{3.5}$$

We refer to this ratio as the FRET-induced donor decay, $\varepsilon_D(t)$, as it quantifies the quenching of the donor by FRET (see **Main Text**, **Fig. 3f**) with rate constant k_{FRET}. $\varepsilon_D(t)$ allows us to directly display the underlying inter-dye distances that correspond to a characteristic time for the FRET species *j* (where *j* can be the species A, B, C and D, respectively) (eq. (3.6)).

$$t_{FRET,j} = \frac{1}{k_{FRET}} = \tau_{D(0),ref} \left(\frac{R_{DA,j}}{R_0(\tau_{D(0),ref})} \right)^6 \tag{3.6}$$

Note that each Förster Radius R_0 has been computed with a specific fluorescence quantum yield of the donor $\Phi_{F,D(0).ref}$ as reference which must be converted to a radiative rate constant by multiplying with the corresponding fluorescence lifetime $\tau_{D(0),ref}$. In this work $\tau_{D(0),ref}$ was 4.0 ns.

The fluorescence decays of the specific donor-only reference and the corresponding FRET samples (DA1, DA2 and DA3, respectively) were analyzed in joint fit as described in detail in ref. [10] to determine the FRET species specific inter-dye distances $R_{DA,j}$. The DA1 Alexa488/647 subpopulation (E_{FRET} > 0.065, dynF, see also **Figure 3f**) and subpopulation with E_{FRET} < 0.065, LF) at 1 mM Mg^{2+} was fitted by eq. (3.1)-(3.4) with a global DOnly decay approximated by a single donor fluorescence lifetime ($\tau_{D(0)}$=4.1 ns) by a model with 3 Gaussian distributed distances and the same half-width σ_{DA}= 6 Å. The fit quality is judged by χ^2_r. The fit results for the subpopulations LF and dynF are collected in the following table:

**subpopulations of DA1**	**$R_{DA,1}$, (Å)**	**x_1**	**$R_{DA,2}$, (Å)**	**x_2**	**$R_{DA,3}$, (Å)**	**x_3**	**χ^2_r**
LF	42	0.28	63	0.49	104	0.23	1.11
dynF		0.40		0.47		0.13	1.07

Further seTCSPC analysis of the fluorescence intensity decay curves for all Alexa488/647 FRET pairs, DA1-3 in 0.5 mM Mg^{2+} with corresponding fits by eq. (3.4) are shown in **Supplementary Fig. 10**. This analysis yielded a good estimate of the FRET parameters of the structural states underlying the dynamic populations (register 1 and 2). Note, that it is difficult to resolve distances of this FRET pair above 90 Å by seTCSPC.

Step 4: Burst-ID FCS

To perform an unbiased check for the presence of exchange kinetics detected by FRET[3], we computed the color correlation functions (auto- (green-green (*G,G*) and red-red (*R,R*))- and cross- (green-red (*G,R*) and red-green (*R,G*)) functions, respectively) for the signal of those bursts, which were selected from the sample as double-labeled species by PIE (**Supplementary Fig. 8a**). These burst-ID cross-correlation functions $G_{G,R}$ and $G_{R,G}$ together with auto-correlation functions $G_{G,G}$ and $G_{R,R}$ were globally fitted by eq. 4.1 with three relaxation times t_{Rj}. To fit the color auto(*i=m*)- and cross(*i≠m*)- correlation functions in a global approach, we have used a set of equations previously presented[5,20]

$$
\begin{aligned}
G_{i=m}(t_c) &= 1+\frac{1}{N_{Br}}\cdot G_{diff}^{(i)}(t_c)\cdot\left[1-B^{(i)}+B^{(i)}\cdot\exp\left(-t_c/t_B\right)+\sum_{j=1}^{n}AC_{Rj}^{(i)}\cdot\left(\exp\left(-\frac{t_c}{t_{Rj}}\right)-1\right)\right] \\
G_{i\neq m}(t_c) &= 1+\frac{1}{N_{CC}}\cdot G_{diff}^{(i,m)}(t_c)\cdot\left[1-CC^{(i,m)}\cdot\sum_{j=1}^{n}X_{Rj}^{(i,m)}\cdot\exp\left(-\frac{t_c}{t_{Rj}}\right)\right]
\end{aligned}
\qquad (4.1)
$$

where t_{Rj} are the relaxation times that correspond to the exchange times between selected color signals ($i=G,R$ and $m=G,R$) with corresponding absolute amplitudes of the auto-correlation function $AC_R^{(i)}$ and the relative normalized amplitudes of the cross-correlation function $CC^{(i,m)}$ with the fractions $X_R^{(i,m)}$. $B^{(i)}$) is the amplitude of an additional bunching term associated to photophysics with the relaxation time t_B in the

measured samples which was globally fitted for the auto-correlation functions. N_{br} is the average number of bright molecules from the color auto-correlation functions in the focus and N_{CC} of the color cross-correlations corresponds to the inverse of the initial amplitude $G_{i,m}(0)$. $G_{diff}^{(x)}(t_c)$ is the apparent diffusion term in the correlation function:

$$G_{diff}^{(x)}(t_c) = \left(1 + \frac{t_c}{t_{diff}^{(x)}}\right)^{-1} \cdot \left(1 + \left(\frac{\omega_0}{z_0}\right)^2 \cdot \frac{t_c}{t_{diff}^{(x)}}\right)^{-\frac{1}{2}}. \tag{4.2}$$

A 3-dimensional Gaussian shaped volume element with parameters ω_0 and z_0 is considered. We assume that $G_{diff}(t_c) = G_{diff}^{(i)}(t_c) = G_{diff}^{(m)}(t_c)$ take the form of eq. (4.2). The selective correlation spectroscopy for the dynF population ($E_{FRET} > 0.065$) of DA1-labeled fibers (Alexa488/647) at 1 mM Mg^{2+} (see **Figure 3g**) by eq. (4.1) are compiled in the following table:

i,m	N_{Br}	z_0/w_0	t_{diff}, [ms]	$B^{(G)}$	t_B, [µs]	$AC_{R1}^{(i)}$ or $X_{R1}^{(i,m)}$	t_{R1}, [µs]	$AC_{R2}^{(i)}$ or $X_{R2}^{(i,m)}$	t_{R2}, [µs]	$AC_{R3}^{(i)}$ or $X_{R3}^{(i,m)}$	t_{R3}, [ms]	$CC^{(i,m)}$
G,G	0.69	70	5.26	0.039	0.192	0.142	2.6	0.089	27.3	0.005	3.14	-
R,R	0.26			0.256		0.183		0.259		0.080		-
G,R	0.72			0		0.168		0.271		0.561		0.42
R,G	0.74			0								0.42

Additional model-free correlation analysis from all FRET vantage points DA1-3 (488/647) at 0.75 mM Mg^{2+} revealed conformational dynamics with at least three relaxation times, thus involving at least four kinetic states (**Supplementary Fig. 11**).

Step 5: Photobleaching and photoblinking analysis

To detect possible photobleaching and photoblinking, we calculated the difference of the burst length in two PIE-channels T_{GX} and T_{RR}, where T_{GX} corresponds to the mean observation time of the photons detected in the donor or acceptor channels after donor excitation (*G*) and T_{RR} corresponds to mean observation time of the red photons after direct excitation of acceptor (*R*) (for details see ref [17]). In case of acceptor photobleaching and/or photoblinking the mean burst time of the acceptor fluorescence emissions is decreased and the mean burst time of donor fluorescence emission is increased simultaneously. This would lead to an increasing T_{GX} and to a decreasing T_{RR}, so that $|T_{GX}-T_{RR}|$ of the analyzed bursts would deviate significantly from zero, if photobleaching and/or photoblinking were present. However, as shown in **Supplementary Fig. 14a**, significant photobleaching and photoblinking was not present under our measurement conditions, because the $|T_{GX}-T_{RR}|$ distribution is symmetric and narrow. Additionally, we checked for the presence of potentially weak

photobleaching and photoblinking processes by applying the macro time filter $|T_{GX}$-$T_{RR}|$< 1 ms threshold criterion for burst selection (**Supplementary Fig. 14b, left panel**). The influence of the presence and absence of this selection criterion on the shape of the FRET efficiency distribution is demonstrated for double labeled bursts of DA1 (Alexa488/647) in 0.5 mM Mg^{2+}. We fitted two FRET efficiency E_{FRET} histograms with and without applied macro time filter (**Supplementary Fig. 14b**) by Photon Distribution Analysis (PDA, see **step 7**) for a 3 ms time window. The obtained FRET distributions and means with (left panel) and without (right panel) burst selection did not significantly differ from each other which proves the absence of marked acceptor photobleaching and photoblinking processes.

Steps 6-11. Establishing a dynamic model for chromatin dynamics

Step 6. Evaluation of kinetic networks between FRET species compatible with experimental data

The detected FRET species, which correspond to structurally meaningful chromatin conformers, form a kinetic network. Using the above presented observations from the various experiments (TIRF, seTCSPC, burst-ID FCS, MFD) kinetic and structural models for chromatin dynamics were formulated (**Supplementary Fig. 15**). The models to be evaluated involved four kinetic states (A-D) in two exchanging dynamic populations (register 1 and 2), corresponding to different tetranucleosome interactions.

In an iterative process, we used dynamic PDA (**step 7**) to refine the parameters and fit the experimental data using the developed kinetic models, followed by model validation (**step 8**). From the obtained, refined parameters, combined with structural molecular modeling (**steps 9-10**), a global model for chromatin conformational change was formulated (**step 11**, **Fig. 5**). The model encompasses a locally dynamic fiber which fluctuates between different tetranucleosome stacking registers on the millisecond timescale. Associations between tetranucleosomes are loose and exchange in the microsecond region. Finally, tetranucleosome open on the millisecond timescale and couple to static locked states, which persist structured over 50-500 ms. The individual **steps 7-11** for this analysis are described below.

Step 7. General description of PDA analysis

Each sample with a specific FRET dye configuration (DA1, DA2 or DA3) was measured at various Mg^{2+} concentrations under single-molecule conditions. The signals of the selected FRET bursts (**Supplementary Fig. 8a**) were split into equal time windows (TW). The FRET efficiency is calculated form the number of photons of donor and acceptor dyes in the prompt time-to-amplitude converter (TAC) channels defined by the donor excitation pulse with a repetition frequency of 32 MHz. In PIE experiments the acceptor excitation laser pulse is delayed by 15.625 ns, which defines the delayed TAC window for computation of the stoichiometry S (see

step 2, **Calculation of donor acceptor distances from fluorescence signal**). For each TW (only full length time windows were used and incomplete pieces at the end of bursts were excluded) the values for the FRET parameters (R_{DA}, E_{FRET}) were calculated as described in **step 2**, **Calculation of donor acceptor distances from fluorescence signal,** and plotted in a 1D frequency histogram with 201 bins (**Supplementary Fig. 16-18**). The fundamental idea in PDA is computing the distribution of the chosen FRET indicator for a given FRET efficiency (or FRET-averaged donor-acceptor distance, $\langle R_{DA}\rangle_E$)[4,14] taking into account photon shot-noise. Due to the flexibility of the dye linker, FRET pairs exhibit a distribution of FRET efficiencies or apparent distances even on rigid molecules, which is caused by distinct acceptor brightnesses[21]. This distance distribution is well approximated by a Gaussian distribution with a half width σ~6 Å.

Calculation of donor acceptor distances from fluorescence signals

The FRET-averaged distance $\langle R_{DA}\rangle_E$ between the dyes could be calculated from the mean FRET efficiency defined in eq. (2.4)

$$\langle R_{DA}\rangle_E = R_0\left(\langle E_{FRET}\rangle - 1\right)^{1/6} \tag{7.1}$$

In this work we calculated $\langle R_{DA}\rangle_E$ directly from the observed intensities and corresponding correction parameters α, β, γ, δ defined in **Step 2**:

$$\langle R_{DA}\rangle_E = R_0\left(\frac{\gamma \cdot I_{G|G}}{I_{R|G} - (\alpha \cdot I_{G|G} + \beta \cdot I_{R|R})}\right)^{1/6} \tag{7.2}$$

using the following FRET pair specifc Förster Radii R_0:

parameter	FRET pair D/A	
	Alexa488/647	Alexa568/647
R_0 [Å]	52	82

Dynamic PDA. Considering the sample DA1 (Alexa488/647) in 1mM Mg^{2+}, we demonstrate the effect of dynamics on the E_{FRET} histograms for two time windows of different length (TW=2ms and TW=5ms, **Supplementary Fig. 14c**). A global fit of both TWs using a joint models with static Gaussian distributions indicates that a model without dynamic exchange terms cannot describe both data sets appropriately, because the exchange dynamics influences the width of the distributions in each TW differently. Therefore we used dynamic PDA[14] in the subsequent analysis, which can describe exchange dynamics comparable to NMR dispersion experiments. For each data set histograms were created for 4 different TWs (2, 3, 4 and 5 ms). All histograms created for Mg^{2+} concentrations (number of [Mg^{2+}] x 4) were globally fitted by the kinetic

models described below. Each FRET species of assembled chromatin was modelled by a Gaussian distribution of distances and was approximated by 51 bins. To describe the histograms of dynamic mixing between two Gaussian distributed FRET species (e.g. species A, species B, ...), this results in $\binom{2}{51}$ = 50*51/2 = 1275 (all possible pairs of distances) dynamic mixing distributions. In contrast, the distributions of two FRET species undergoing dynamic mixing are approximated by 201 bins. Then, for each pair of interconverting Gaussians distributed species (e.g. dyn A-B, dyn B-C, ...), the shot-noise limited total histogram could be calculated as a sum of all 1275*201 = 256275 shot-noise limited FRET parameter distributions. We have shown that shot-noise limited FRET parameter histograms from Gaussian distributed distances (R_{mean}, σ) and the sum of shot-noise limited FRET parameter histograms of two fixed distances R_{mean}- σ and R_{mean}+ σ are very similar [14]. Thus, in order to simulate dynamics between two Gaussian distributed species and to reduce computational cost, the model distribution can be approximated with the sum of two dynamic distributions between ($R1_{mean}$- $\sigma1$ and $R2_{mean}$- $\sigma2$) and ($R1_{mean}$+ $\sigma1$ and $R2_{mean}$+ $\sigma2$) (**Supplementary Fig. 14d**).

MFD data were then globally fitted using dynamic PDA and assuming a kinetic model[14]. To satisfy the observations of coexisting dynamic and quasi-static molecules in smTIRF experiments, each dynamic PDA model assumed the co-existence of molecules existing in a number of individual FRET species showing no dynamics on the MFD timescale (t_R > 50 ms, static fraction, stat A, stat B, …), with populations of molecules which exchange between FRET species (dynamic fractions, dyn A-B, dyn B-C, …). Secondly, we assumed the inter-dye distances in the basic FRET species (A, B, C, D) to be invariant to Mg^{2+}. Thus, a sum of Gaussian distributed FRET species (static fractions, R_{DAi}) and dynamically mixing Gaussian distributed FRET species pairs (dyn i-j) corresponding to the selected kinetic model (see list of trial models in **Supplementary Fig. 15**) was used to globally fit the group of histograms for each FRET dye configuration over all Mg^{2+} concentrations (**Fig. 5a-c** and final models in **Supplementary Fig. 16-18**). Thirdly, the dynamics of exchanging molecules was described by models with a series of two-state kinetic exchange terms connecting the quasi-static populations. Final models are shown in **Fig. 5d-f** and **Supplementary Fig. 16-18**. Importantly, global fits were employed to evaluate the Mg^{2+} dependencies, assuming a linear relationship between the logarithms of the rate constants and the ionic strength:

$$\log(k_{ij}) = m \cdot [Mg^{2+}] + k_{ij,0}, \qquad (7.3)$$

similar to observations in protein folding[22].

Step 8: Validation of kinetic models.

Based on the model-free FCS analysis (**Supplementary Fig. 11**), yielding 3 relaxation times, at least four kinetically relevant species are expected. As our dye configurations (DA1-3) are not equally sensitive to all structural exchanges processes and states (**Fig. 4a**) we tested models containing 3 or 4 states for each dye configuration in the dynamic PDA analysis, employing various connectivities. For each configuration DA1-3 different kinetic models were evaluated based on a number of criteria:

i. Physical meaningful connectivity of species,

ii. Minimal number of species,

iii. Cutoff for rate constants (10^6 s^{-1} > k_{ij} > 10^2 s^{-1}) and m-values (-25 < m < 25 (see equation (7.3)); assuming a change of less than ±100-fold in each rate constant within the tested Mg^{2+} concentrations),

iv. Stable fit results over the different Mg^{2+} concentration,

v. Acceptable goodness of fit (χ^2_r < 1.4),

vi. Consistency with models from other FRET vantage points (DA1, DA2 and DA3),

vii. Transitions between FRET species which are structurally meaningful.

A number of different models were tested and based on criteria *i-vi* (**Supplementary Fig. 15**). The models shown in Fig. 3d were deemed to be the most probable to describe the experimental data. Finally, from the static and dynamic fractions, weighted by the associated rate constants, the relative populations of each state were calculated (**Fig. 5g-i**) according to $P_i = P_i^s + P_{ij}^d \cdot k_{ij} / (k_{ij} + k_{ji})$, where P_i denotes the population of state i, P^s_i is the static fraction, P^d_{ij} is the dynamic fraction between states i and j and k_{ij}, k_{ji} are the associated rate constants.

Steps 9-11. Assignment of the states

For a structural interpretation of the detected inter-dye distances from MFD and PDA, we determined the uncertainties in our analysis and subsequently applied structural modeling, using both available structures and coarse grained modeling, in combination with modeling of the conformational distributions of the dyes.

Step 9: Relating measured R_{DA} distances to structural models of compact chromatin states

Here, first we define the uncertainties in the measured parameters, followed by the construction of molecular models for the compact states.

Determination of the uncertainties for structural modeling. As a first test for the suitability of the dyes Alexa488 and Alexa647 for an accurate structural analysis based on FRET data, we checked the fluorescence lifetimes of the donor-only and acceptor samples and time-resolved anisotropies *r(t)* using the FRET samples identified by PIE. The results (see **summaries 9.1-2** of donor dye properties, **summaries 9.3-4** of acceptor dye properties below) indicate that there is no strong quenching as compared to the free dyes in solution and that all dyes are sufficiently mobile at these positions. Anisotropy decays were analyzed by eq. (9.1).

$$r(t) = \sum_i r_i \exp(-t/\rho_i) \; and \; \sum_i r_i = r_0 \qquad (9.1)$$

Here r_i denotes the depolarization fractions related to order parameters, and ρ_i the corresponding depolarization times mainly by dye rotation. In the *r(t)* analysis we applied the fundamental anisotropies r_0 = 0.38 for Alexa488 and Alexa647, respectively. We used the amplitude of the longest depolarization time r_i to approximate the residual anisotropy r_{inf} for computing the dye and position specific fraction of trapped dyes using eq. (9.2).

$$x_{trapped} = r_{inf} / r_0 \qquad (9.2)$$

The fraction of trapped dyes is needed to parametrize the contact volume for improving the accuracy of the estimated spatial dye density in ACV simulations described in **step 9**, section **FRET positioning and screening calculations** below.

Summary of dye properties of the donor Alexa488. In the tables below the fluorescence lifetimes and anisotropy contributions of this donor dye are compiled.

Summary 9.1. Fluorescence lifetimes of the donor dye Alexa488

**Species**	**τ (ns)**
**DA1**	
Average	4.2
Std, Δ	< 0.1
**DA2**	
Average	4.1
Std, Δ	< 0.1
**DA3**	
Average	4.1
Std, Δ	< 0.1
**Average of DA1-3**	
Average	4.1
Std, Δ	< 0.1

Summary 9.2. Fluorescence anisotropy and rotational correlation times of the donor dye Alexa488

Species	ρ_1 (ns)	ρ_2 (ns)	ρ_3 (ns)	ρ_4 (ns)	r_1	r_2	r_3	$r_{04}=r_{inf,D}$
**DA1**								
Average [a]	< 0.3	1.1	4.8	> 40	0.162	0.052	0.087	0.080
Std, Δ		0.2	0.9		0.014	0.011	0.014	0.012
$x_{trapped}$								**0.210**
**DA2**								
Average [a]	< 0.3	0.6	5.0	> 40	< 0,01	0.196	0.102	0.083
Std, Δ		0.1	1.1			0.069	0.030	0.009
$x_{trapped}$								**0.218**
**DA3**								
Average [a]	< 0.3	0.6	3.3	> 40	0.032	0.199	0.092	0.057
Std, Δ		0.03	0.5		0.007	0.012	0.009	0.003
$x_{trapped}$								**0.149**

[a] The fit values with using eq. (9.1) are averages for measurements at 0.0, 0.5, 1.0 and 4 mM Mg^{2+}.

Summary of acceptor dye properties. In the tables below the fluorescence lifetimes and anisotropy contributions of the acceptor dye Alexa647 is summarized. In practice, as all cyanine based dyes, Alexa647 can have several dye populations in distinct environments with specific brightnesses when coupled to biomolecules referred to as acceptor heterogeneity, A_{het}. This typical behavior is also seen in nucleosome arrays (see Summary 9.3). In this case a fixed DA distance is usually not sufficient to describe FRET species, and a Gaussian distance distribution with a mean apparent distance $\langle \tilde{R} \rangle$ and an apparent distribution half width (hw_{app}) has to be used instead. As shown by Kalinin and colleagues[21] $\langle \tilde{R} \rangle$ is slightly biased towards longer distances as compared to $\langle R_{\mathrm{DA}} \rangle_E$ (eq. (9.3)).

$$\langle \tilde{R} \rangle = \langle R_{\mathrm{DA}} \rangle_E \langle \Phi_{\mathrm{FA}} \rangle^{1/6} \langle (\Phi_{\mathrm{FA}})^{-1/6} \rangle \quad (9.3)$$

where Φ_{FA} is the acceptor fluorescence quantum yield. Note that the fraction of fluorescent trans states a (usually a = 0.8) cancels out in eq. (9.3)-(9.4). In this work, the correction factors $\langle \Phi_{\mathrm{FA}} \rangle^{1/6} \langle \Phi_{\mathrm{FA}}^{-1/6} \rangle$ are very close to unity (Summary 9.3) and thus can be disregarded for the calculation of interdye distances (i.e. in this work $\langle \tilde{R} \rangle \cong \langle R_{\mathrm{DA}} \rangle_E$). Applying the rules for error propagation for the function $\tilde{R}$ (Φ_{FA}), one obtains also an relation for the variance and half width (σ) of the apparent DA distance (eq. 9.4).

$$\sigma(\tilde{R}) / R_{\mathrm{DA}} = \langle \Phi_{\mathrm{FA}} \rangle^{1/6} \left[\mathrm{var}(\Phi_{\mathrm{FA}}^{-1/6}) \right]^{1/2} \quad (9.5)$$

The fact that relative experimental half widths (σ/R_{DA}) (**Supplementary Figures 16-18,** σ/R_{DA}(DA1) = 0.1, σ/R_{DA}(DA2) = 0.13, σ/R_{DA}(DA3) = 0.06) are much broader than the values caused by acceptor heterogeneity

(Summary 9.4) may indicate significant heterogeneity of the nucleosome arrays which would be actually not surprising. Note that the difference is the smallest (less than factor 2) for DA3.

Summary 9.3. Fluorescence lifetimes and other dye parameters of the acceptor dye Alexa647 with a= 0.8.

**Species**	τ_1 **(ns)**	τ_2 **(ns)**	x_1	x_2	$\langle\tau_A\rangle_x$ **(ns)**	$a\ \Phi_{FA}$ **[a,b]**	A heterogeneity, A_{het} $\langle\Phi_{FA}\rangle^{1/6}\langle(\Phi_{FA})^{-1/6}\rangle$	σ/R_{DA}
**DA1**								
Average [b]	1.38	1.68	0.55	0.45	1.43	0.390	1.0001	0.005
Std, Δ	0.04	0.31	0.37	0.37	0.01	0.002	< 0.0001	0.001
**DA2**								
Average	0.92	1.49	0.32	0.68	1.32	0.362	1.0038	0.035
Std, Δ	0.10	0.005	0.18	0.18	0.06	0.015	0.0007	0.005
**DA3**								
Average	0.90	1.49	0.36	0.64	1.28	0.350	1.0051	0.040
Std, Δ	0.09	0.06	0.12	0.12	0.01	0.002	0.0003	0.002
**Average of DA1-3**								
Average					1.34	0.368		
Std, Δ					0.06	0.017		

[a] We used the reference value of Cy5 labeled dsDNA with $\langle\tau_A\rangle_x$ = 1.17 ns and Φ_{FA} = 0.4 which was measured with low irradiances at a steady state fluorescence spectrometer, i.e. *a*=0.
[b] The fit values are averages for measurements at 0.0, 0.5, 1.0 and 4 mM Mg^{2+} using a fit with a series of exponentials $f_A(t) = \sum_i x_i \exp(-t/\tau_i)\ and\ \sum_i x_i = 1$.

Summary 9.4. Fluorescence anisotropy and rotational correlation times of the acceptor dye (Alexa647)

**Species**	ρ_1 **(ns)**	ρ_2 **(ns)**	ρ_3 **(ns)**	r_1	r_2	$r_3=r_{inf,A}$
**DA1**						
Average [a]	< 0.3	1.6	10.2	0.063	0.054	0.263
Std, Δ		0.7	0.4	0.007	0.026	0.020
$x_{trapped}$						**0.692**
**DA2**						
Average [a]	< 0.3	0.9	13.7	0.072	0.056	0.252
Std, Δ		0.6	4.1	0.003	0.019	0.018
$x_{trapped}$						**0.663**
**DA3**						
Average [a]	< 0.3	1.0	9.9	0.081	0.090	0.209
Std, Δ		0.3	0.03	0.007	0.013	0.016
$x_{trapped}$						**0.549**

[a] The fit values with using eq. (9.1) are averages for measurements at 0.0, 0.5, 1.0 and 4 mM Mg^{2+}.

Determination of uncertainties in measured R_{DA} distances

The uncertainty in the measured R_{DA} distances, used for structural modeling, is obtained by determining the individual uncertainties of all quantities separately, and then propagating them towards an uncertainty in the distance. Considering the DA distance, R_{DA}, two main factors determine the uncertainty, ΔR_{DA} in this study: (1) the precision (noise) of the measurement, Δ_{noise} and (2) the uncertainty of the calibration, Δ_{cal}. The total uncertainty of the distance, ΔR_{DA}, is estimated by combining these error sources. With the assumption that the contributions follow a normal distribution, ΔR_{DA} is given by:

$$\Delta R_{DA} = \left[\Delta_{\text{noise}}^{2} + \Delta_{\text{cal}}^{2}\right]^{1/2} \tag{9.6}$$

The distance, R_{DA}, can be expressed as a function of experimental observable fluorescence intensities and correction and conversion parameters (see eq. 7.2). Thus Δ_{cal}^{2} can be expanded as[10]

$$\Delta R_{DA} = \left[\Delta_{noise}^{2} + \left(\Delta_{B_{G|G}}^{2} + \Delta_{B_{R|G}}^{2} + \Delta_{\alpha}^{2} + \Delta_{\beta}^{2} + \Delta_{\gamma}^{2} + \Delta R_{0}^{\ 2}\right)\right]^{1/2} \tag{9.7}$$

Calibration contributions to the uncertainty, $\Delta R_{DA,cal}$. All equations used to compute the contributions $\Delta R_{DA,cal}$. were described in detail by Peulen et al. (eqs. 39-46 in ref. [10]).

Contributions to the uncertainty ΔR_0. The overall uncertainty for the Förster radius, ΔR_0, is estimated by the uncertainties of the local refractive index, n, the exact donor fluorescence quantum yield, $\Phi_{F,D}$, spectral overlap integral, J, and the FRET orientations factor, κ^2, ref. [11] (eq. (9.8)).

$$\Delta R_0\left(n^{-4}, \Phi_{F,D}, J, \kappa^2\right) = \sqrt{\Delta R_0(n)^2 + \Delta R_0\left(\Phi_{F,D}\right)^2 + \Delta R_0(J)^2 + \Delta R_0(\kappa^2)^2} \sim 0.08 - 0.09 \cdot R_0 \tag{9.8}$$

Contributions to the uncertainty $\Delta R_{DA}(R_{DA})$ by noise. We have to determine the precision of the dynamic PDA fits, $\Delta R_{\text{DA}}(R_{\text{DA}})$ caused by statistical noise. Here, we performed a subsampling analysis, where the dynamic PDA fit procedure was repeated three times using a 70% subsample of the total dataset. The standard deviations from these three fits are reported in **Supplementary Figures 16-18**, and determine the precision of our fitting procedure. The overall precision in R_{DA}, $\Delta R_{\text{DA}}(R_{\text{DA}})$, from dynamic PDA is reported in **Supplementary Table 7**: 2% (DA1 and DA3) and 3% (DA2).

Contributions to the total uncertainty $\Delta R_{DA}(R_{DA})$. The individual errors are listed in **Supplementary Table 7**. They are then propagated using eq. (9.7), to estimate the total uncertainty of the determined distances. Together, these analyses result in a total uncertainty for R_{DA} for DA1 of 9%, for R_{DA} for DA2 of 9% and R_{DA} for DA3 of 8% (**Supplementary Table 7**).

Model building. We built models using the cryo-EM structure of a 12-mer nucleosomal array with 177 bp nucleosome repeat length[5]. We then modeled the accessible contact volume (ACV) for dyes in the DA1, DA2 or DA3 configuration and employed these distance distributions to calculate an average, conformation-weighted inter-dye distance (see below, **FRET positioning and screening calculations**). Importantly, we considered two possible fiber structures: The 12-mer array could exist as a stack of three tetranucleosome (TN) units (TN1(N1-N4); TN2(N5-N8); TN3(N9-N12), 4-4-4, register 1) as observed in the cryoEM structure (see Fig. 1a). Alternatively, tetranucleosomes could stack in a different register (TN1(N3-N6); TN2(N7-N10), with four unstacked nucleosomes at both ends, 2-4-4-2, register 2). This would put the DA1-3 dye pairs into neighboring tetranucleosomes. Finally, if the nucleosome-nucleosome interactions are local and fiber compaction is not fully cooperative, both registers are expected to be populated. We thus produced models for both registers and calculated the expected inter-dye distances for DA1-3 in register 1 and 2 (**Supplementary Fig. 12 and Supplementary Table 8**). The observed deviations for DA2 can be rationalized by rotational motions between two nucleosomes (see **Supplementary Fig. 13 e,f**). A "clamshell" motion by ~10° would be sufficient to explain the experimental data of DA2 (488/647). Note that DA1 is relatively insensitive to these motions.

FRET positioning and screening calculations. The dye distribution was modeled by the accessible contact volume approach (ACV)[4] which is similar to the accessible volume (AV)[6], but additionally defines an area close to the surface as contact volume. Here donor and acceptor fluorophores are approximated by a ellipsoid with an empirical radius $R_{dye(i)}$ and where central atom of the dye is connected via flexible linkage with effective length L_{link} and width w_{link} to the C_5 atom in the dT nucleotide. All geometric parameters for the dyes were: Alexa488: L_{link} =20 Å, w_{link}=4.5 Å, $R_{dye(1)}$=5 Å, $R_{dye(2)}$=4.5 Å, $R_{dye(3)}$=1.5 Å, Alexa568: L_{link} =22 Å, w_{link}=4.5 Å, $R_{dye(1)}$=7.8 Å, $R_{dye(2)}$=1.9 Å, $R_{dye(3)}$=1.5 Å, Alexa647: L_{link} =22 Å, w_{link}=4.5 Å, $R_{dye(1)}$=11 Å, $R_{dye(2)}$=3 Å, $R_{dye(3)}$=1.5 Å (**Supplementary Fig. 12**). In the ACV model the part of AV which is closer than 3 Å from the macromolecular surface (referred to as contact volume) is defined to have a distinct spatial dye density ρ_{dye}. In this model, where a dye freely diffuses within the AV and its diffusion is hindered close to the surface, the spatial density ρ_{dye} along R is approximated by a step function: $\rho_{dye}(R < 3\ \text{Å}) = \vartheta_{CV,dye} \cdot \rho_{dye}(R \geq 3\ \text{Å})$. Here $\vartheta_{CV,dye}$ corresponds to the relative dye density in the contact volume relative to outer volume. $\vartheta_{CV,dye}$ is adjusted such that fraction of trapped dyes, determined by the residual anisotropy (see table above) is met. Note that $\vartheta_{CV,dye}$ is specific for each ACV because the shape, size, and surface area to the nucleosome varies slightly for each dye position. In the following table, we indicate the fraction of trapped dye and the dye density in the contact volume relative to the outer volume $\vartheta_{CV,dye}$ for DA1-3:

Summary 9.5. Parameters for modeling the contact volume in the ACV simulations

	**Donor register 1**	**Donor register 2**	**Acceptor register 1**	**Acceptor register 2**
**DA1**				
$x_{trapped}$ [a]	0.210		0.692	
$\vartheta_{CV,dye}$	0.1	0.3	1.6	3.1
**DA2**				
$x_{trapped}$	0.218		0.663	
$\vartheta_{CV,dye}$	0.3	0.1	2.1	2.3
**DA3**				
$x_{trapped}$	0.149		0.549	
$\vartheta_{CV,dye}$	0.2	0.2	1.6	1.6

[a] computed by eq. 9.2 with values from the Summary 9.2 (donor) and Summary 9.4 (acceptor).

Step 10: Structural models of open and dynamic states

To model the unfolded and open chromatin state, we further resorted to computational modeling. Specifically, we performed Monte Carlo simulations of an established coarse-grained model of the chromatin fiber[7,23] (see below **Coarse grained simulations**) to generate an ensemble of open chromatin conformations in the same temperature and salt conditions as the experiments, but in the absence of inter-nucleosome stacking interactions between the H4 histone tail and the acidic patch. From this larger ensemble of conformations, a hundred relatively uncorrelated structures were picked and used to build all-atom models of the chromatin configurations (**Supplementary Fig. 13a**). We then measured inter-dye distances for all nucleosomes in these structures for DA1-3 and produced distance distribution histograms (**Supplementary Fig. 13b,c,d**). These histograms showed that expected peak inter-dye distances were 110 Å (and a smaller fraction of structures with 190 Å) for DA1, 80 Å and 120 Å for DA2 and 90 Å for DA3. These distances match distances expected for states D in the PDA (**Fig. 4a**).

Finally, to understand the intra-tetranucleosome dynamics observed for DA2 (**Fig. 4a,b**) we employed the tetranucleosome X-ray structure[13] to test how structural distortions affect inter-dye distances for DA1 and DA2 (**Supplementary Fig. 13e,f**) and DA3 (**Supplementary Fig. 13g**). DA2 was found to be more sensitive to tetranucleosome distortions, and distances observed for state C could be modeled by a 30° change in the tetranucleosome interaction angle (**Supplementary Fig. 13e**) or by a 30° rotation of one nucleosome relative to its neighbor (**Supplementary Fig. 13f**). Importantly, these conformations still allow interactions at the H2B and H2A four-helix bundle[5] to persist. To illustrate the effect of nucleosome structural motions on each of FRET dye configurations (DA1, DA2, DA3), we plot FRET-average inter-dye distance as a function of the motion coordinate (**Supplementary Fig. 13e,f**). We used tetranucleosome structural models[13] as a starting point for our illustrations. First we tested, how the DA1 and DA2 inter-dye distances change with respect to the

clamshell-like opening angle between the two nucleosome units (N5, N7, **Supplementary Fig. 13e**). To define the clamshell rotation coordinate, we chose an axis going through the phosphorous atom of the unit N7, chain B, residue 55 and the phosphorous atom of the unit N7, chain A, residue -30. Thus, clamshell motion is the rotation of the unit N7 around the specified axis with the origin at the phosphorous atom of N7, chain B, residue 55. Second, we tested the DA1 and DA2 distance change with respect to the in-plane nucleosome rotation. To define this second rotational motion coordinate we chose the rotation axis between the centers of mass of the nucleosome units N5 and N7. Thus N7 is rotated around the specified axis with the origin at the center of mass of N7.

As the result we have observed that DA2 distance senses nucleosome clamshell motion while DA1 does not (**Supplementary Fig. 13e**). $\langle R_{DA}\rangle_E$ for DA2 changes from 69 Å to 48 Å in the angular range of -30° to 0°. DA1 is not sensitive to this motion and varies only from 47 to 50 Å. In the case of in-plane rotation, $\langle R_{DA}\rangle_E$ for DA2 drops from 70 Å to 50 Å, when angle ranges from -30° to 30°. $\langle R_{DA}\rangle_E$ for DA1 is also sensitive to this motion and shows an increase of $\langle R_{DA}\rangle_E$ from 45 Å to 58 Å.

Coarse grained simulations

The 12-nucleosomes chromatin fibers with 177bp repeats (~30bp linker DNAs) were treated at a coarse-grained resolution using a mesoscopic model developed and validated by Arya and Schlick[23,24]. According to this model, each nucleosome core (histone octamer plus wound DNA) is treated as a rigid body with an irregular surface described by 300 charged beads; the linker DNAs are treated as charged bead-chains with each bead representing a 3 nm-long segment of double-stranded DNA; and the histone tails (N termini of H2A, H2B, H3, and H4 and C termini of H2A) are also treated as charged bead-chains, where each bead represents five amino acid residues. The core, linker, and tail beads are assigned excluded volume potentials, to prevent them from overlapping with each other, and charges, to reproduce the electrostatic field of their corresponding atomistic counterpart at the specified salt concentration. The linker DNAs are assigned an intramolecular force field to reproduce experimentally obtained bending and torsional rigidity of DNA, and the histone tails are assigned an intramolecular force field to reproduce the configurational properties of atomistic histone tails. In this study, the nucleosome entry/exit angle was set to 130°, compatible with the trajectory of linker DNA in the tetranucleosome structure of Song et al.[5], and the monovalent salt concentration was set to 50 mM. The effects of Mg^{2+} were treated phenomenologically, with suitably modified Debye length and persistence length of the linker DNA, as described elsewhere[23].

To generate an equilibrium ensemble of fiber conformations at 293 K, we used a tailored Monte Carlo simulation approach as described elsewhere[24]. Briefly, the simulations employed four Monte Carlo “moves”: global pivot rotation of the end portions of the fiber about a randomly picked nucleosome core or linker DNA

bead, local translation and rotation of a randomly picked linker DNA bead or nucleosome core, and configurational bias regrowth of a randomly picked histone tail. The simulations were performed for 40 million steps, with the above four Monte Carlo moves implemented at a relative frequency of 0.2: 0.1: 0.1: 0.6, respectively. We picked a total of 100 uncorrelated fiber conformations from the simulated ensemble, which were then used to generate the corresponding atomistic models of the fiber (**Supplementary Fig. 13a-d**).

Step 11: Final model and its validation - A unified model of chromatin dynamics

Based on the analyses presented above (**steps 1-10**) we formulated a unified model for chromatin dynamics (**Fig. 6**). The model encompasses two dynamic populations, corresponding to two tetranucleosome registers (register 1 and 2). From dynPDA of DA1 – 3, ranges for the exchange rate constants were determined and are given in Fig. 4i. The presented model is well supported by the whole of the experimental data and is corroborated by matching results from different analyses yielding FRET efficiency states, dynamics rate constants and populations (**Steps 2 - 4**) and dynamic PDA (**Step 6**).

Supplementary References

1. Robinson, P.J., Fairall, L., Huynh, V.A. & Rhodes, D. EM measurements define the dimensions of the "30-nm" chromatin fiber: evidence for a compact, interdigitated structure. *Proc. Natl. Acad. Sci. U.S.A.* **103**, 6506-11 (2006).
2. Li, F., Allahverdi, A., Yang, R., Lua, G.B.J., Zhang, X., Cao, Y., Korolev, N., Nordenskiöld, L. & Liu, C.-F. A direct method for site-specific protein acetylation. *Angew. Chem. Int. Ed. Engl.* **50**, 9611-14 (2011).
3. Felekyan, S., Sanabria, H., Kalinin, S., Kühnemuth, R. & Seidel, C.A.M. Analyzing Forster resonance energy transfer with fluctuation algorithms. *Methods Enzymol.* **519**, 39-85 (2013).
4. Dimura, M., Peulen, T.O., Hanke, C.A., Prakash, A., Gohlke, H. & Seidel, C.A.M. Quantitative FRET studies and integrative modeling unravel the structure and dynamics of biomolecular systems. *Curr. Opin. Struct. Biol.* **40**, 163-185 (2016).
5. Song, F., Chen, P., Sun, D., Wang, M., Dong, L., Liang, D., Xu, R.M., Zhu, P. & Li, G. Cryo-EM study of the chromatin fiber reveals a double helix twisted by tetranucleosomal units. *Science* **344**, 376-80 (2014).
6. Kalinin, S., Peulen, T., Sindbert, S., Rothwell, P.J., Berger, S., Restle, T., Goody, R.S., Gohlke, H. & Seidel, C.A.M. A toolkit and benchmark study for FRET-restrained high-precision structural modeling. *Nat. Methods* **9**, 1218-25 (2012).
7. Arya, G., Zhang, Q. & Schlick, T. Flexible histone tails in a new mesoscopic oligonucleosome model. *Biophys. J.* **91**, 133-50 (2006).
8. Kilic, S., Bachmann, A.L., Bryan, L.C. & Fierz, B. Multivalency governs HP1alpha association dynamics with the silent chromatin state. *Nat. Commun.* **6**, 7313 (2015).
9. Nishibuchi, G., Machida, S., Osakabe, A., Murakoshi, H., Hiragami-Hamada, K., Nakagawa, R., Fischle, W., Nishimura, Y., Kurumizaka, H., Tagami, H. & Nakayama, J. N-terminal phosphorylation of HP1alpha increases its nucleosome-binding specificity. *Nucleic Acids Res.* **42**, 12498-511 (2014).
10. Peulen, T.O., Opanasyuk, O. & Seidel, C.A.M. Combining Graphical and Analytical Methods with Molecular Simulations To Analyze Time-Resolved FRET Measurements of Labeled Macromolecules Accurately. *J. Phys. Chem. B* **121**, 8211-8241 (2017).

11. Hellenkamp, B. et al. Precision and accuracy of single-molecule FRET measurements - a worldwide benchmark study. *arXiv:1710.03807 [q-bio.QM]* (2017).
12. Sindbert, S., Kalinin, S., Nguyen, H., Kienzler, A., Clima, L., Bannwarth, W., Appel, B., Muller, S. & Seidel, C.A.M. Accurate distance determination of nucleic acids via Forster resonance energy transfer: implications of dye linker length and rigidity. *J. Am. Chem. Soc.* **133**, 2463-80 (2011).
13. Schalch, T., Duda, S., Sargent, D.F. & Richmond, T.J. X-ray structure of a tetranucleosome and its implications for the chromatin fibre. *Nature* **436**, 138-41 (2005).
14. Kalinin, S., Valeri, A., Antonik, M., Felekyan, S. & Seidel, C.A.M. Detection of structural dynamics by FRET: a photon distribution and fluorescence lifetime analysis of systems with multiple states. *J. Phys. Chem. B* **114**, 7983-95 (2010).
15. Sisamakis, E., Valeri, A., Kalinin, S., Rothwell, P.J. & Seidel, C.A.M. Accurate single-molecule FRET studies using multiparameter fluorescence detection. *Methods Enzymol.* **475**, 455-514 (2010).
16. Fries, J.R., Brand, L., Eggeling, C., Kollner, M. & Seidel, C.A.M. Quantitative identification of different single molecules by selective time-resolved confocal fluorescence spectroscopy. *J. Phys. Chem. B* **102**, 6601-6613 (1998).
17. Kudryavtsev, V., Sikor, M., Kalinin, S., Mokranjac, D., Seidel, C.A.M. & Lamb, D.C. Combining MFD and PIE for accurate single-pair Forster resonance energy transfer measurements. *ChemPhysChem* **13**, 1060-78 (2012).
18. Lee, N.K., Kapanidis, A.N., Wang, Y., Michalet, X., Mukhopadhyay, J., Ebright, R.H. & Weiss, S. Accurate FRET measurements within single diffusing biomolecules using alternating-laser excitation. *Biophys. J.* **88**, 2939-53 (2005).
19. Vopel, T., Hengstenberg, C.S., Peulen, T.O., Ajaj, Y., Seidel, C.A.M., Herrmann, C. & Klare, J.P. Triphosphate induced dimerization of human guanylate binding protein 1 involves association of the C-terminal helices: a joint double electron-electron resonance and FRET study. *Biochemistry* **53**, 4590-600 (2014).
20. Felekyan, S., Kalinin, S., Sanabria, H., Valeri, A. & Seidel, C.A.M. Filtered FCS: species auto- and cross-correlation functions highlight binding and dynamics in biomolecules. *ChemPhysChem* **13**, 1036-53 (2012).
21. Kalinin, S., Sisamakis, E., Magennis, S.W., Felekyan, S. & Seidel, C.A.M. On the origin of broadening of single-molecule FRET efficiency distributions beyond shot noise limits. *J. Phys. Chem. B* **114**, 6197-206 (2010).
22. Song, B., Cho, J.H. & Raleigh, D.P. Ionic-strength-dependent effects in protein folding: analysis of rate equilibrium free-energy relationships and their interpretation. *Biochemistry* **46**, 14206-14 (2007).
23. Arya, G. & Schlick, T. A tale of tails: how histone tails mediate chromatin compaction in different salt and linker histone environments. *J. Phys. Chem. A* **113**, 4045-59 (2009).
24. Grigoryev, S.A., Arya, G., Correll, S., Woodcock, C.L. & Schlick, T. Evidence for heteromorphic chromatin fibers from analysis of nucleosome interactions. *Proc. Natl. Acad. Sci. U.S.A.* **106**, 13317-22 (2009).
